# A High-Throughput Fluorescent Turn-On Assay for Inhibitors of DHHC Family Proteins

**DOI:** 10.1021/acschembio.2c00193

**Published:** 2022-07-11

**Authors:** Tian Qiu, Saara-Anne Azizi, Noah Brookes, Tong Lan, Bryan C. Dickinson

**Affiliations:** † Department of Chemistry, The University of Chicago, Chicago, Illinois 60637, United States; ‡ Medical Scientist Training Program, Pritzker School of Medicine, The University of Chicago, Chicago, Illinois 60637, United States

## Abstract

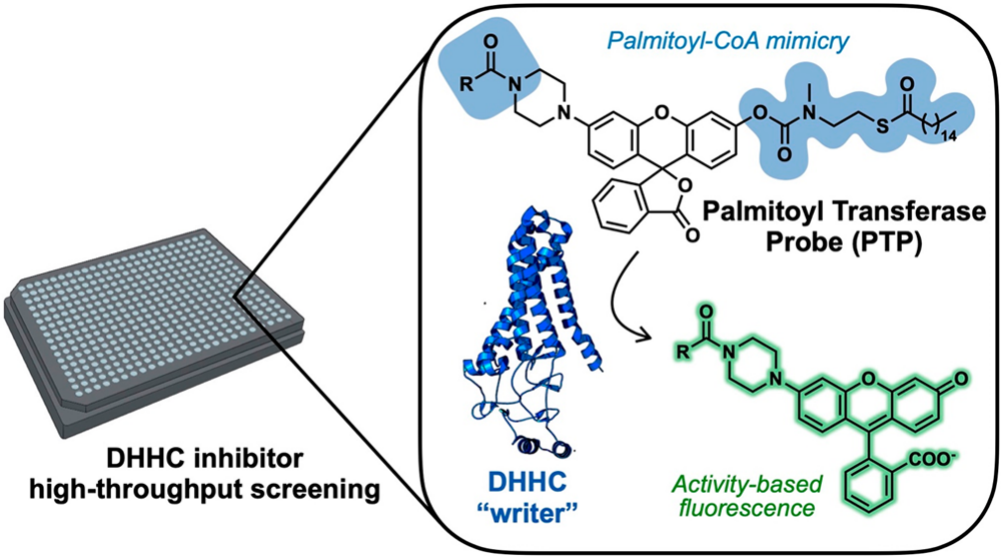

As the “writer” enzymes of protein *S-*acylation, a dynamic and functionally significant post-translational modification (PTM), DHHC family proteins have emerged in the past decade as both key modulators of cellular homeostasis and as drivers of neoplastic, autoimmune, metabolic, and neurological pathologies. Currently, biological and clinical discovery is hampered by the limitations of existing DHHC family inhibitors, which possess poor physicochemical properties and off-target profiles. However, progress in identifying new inhibitory scaffolds has been meager, in part due to a lack of robust in vitro assays suitable for high-throughput screening (HTS). Here, we report the development of palmitoyl transferase probes (PTPs), a novel family of turn-on pro-fluorescent molecules that mimic the palmitoyl-CoA substrate of DHHC proteins. We use the PTPs to develop and validate an assay with an excellent Z′-factor for HTS. We then perform a pilot screen of 1687 acrylamide-based molecules against zDHHC20, establishing the PTP-based HTS assay as a platform for the discovery of improved DHHC family inhibitors.

## Introduction

Protein lipidation via reversible thioester linkages at cysteine residues, termed *S-*acylation, is an abundant and influential post-translational modification (PTM).^1^ Also referred to as *S-*palmitoylation, given the frequent occurrence of the C16:0 lipid, *S*-acylation occurs on diverse protein substrates, from scaffolding and signaling proteins to ion channels and receptors, and has diverse impacts on the target protein, including modulating activity, stability, interactions, and subcellular trafficking.^2,3^*S*-acylation is dynamic and enzymatically regulated, with its installation mediated by 23 zDHHC (zinc finger Asp-His-His-Cys) domain-containing “writer” protein acyltransferases (PATs) and removal mediated by serine hydrolase family “eraser” acyl protein thioesterases (APTs), including APT1/2, PPT1/2, ABHD17A/B/C, and ABHD10.^4−9^

DHHCs and APTs regulate the cycle of proteome-wide acylation/deacylation and subsequent cell signaling, impacting cellular homeostasis and function.^10^ Moreover, in recent years, both DHHCs and APTs have emerged as targets for the mitigation of human pathologies. For example, the *S*-palmitoylation and *S*-depalmitoylation of N-Ras by zDHHC9 and ABHD17, respectively, regulate its activation state and signal propagation and, therefore, could be targeted in N-Ras-dependent cancers.^8,11^ Perturbing the *S*-palmitoylation cycle of STAT3 by inhibiting either zDHHC7 or APT2 activity can preclude T_H_17 cell differentiation and reduce symptoms of inflammatory bowel disease.^12^ In addition, zDHHC20 activity has been implicated in cellular transformation and lung tumorigenesis via regulation of epidermal growth factor receptor (EGFR) signaling, while zDHHC20 is upregulated in colorectal cancer.^13,14^ Thus, the disruption of *S*-acylation represents an emerging strategy for the treatment of disease.

While potent pan-active and isoform-specific *S-*deacylase (APT) inhibitors exist, parallel tools for the DHHC-PATs —much less molecules with clinical potential remain scant. 2-Bromopalmitate (2BP) is the most commonly used DHHC inhibitor, but low potency, high cytotoxicity, and poor selectivity significantly curtail its applications.^15^ Our laboratory recently developed an acrylamide-based DHHC inhibitor, cyanomyracrylamide (CMA), which has decreased cytotoxicity and an altered reactivity profile, compared to 2BP. However, similar to 2BP, CMA is a lipid-based molecule with limited selectivity that targets a broad spectrum of DHHC family proteins, making it unsuitable for probing the biology of individual DHHCs.^16^

A central challenge in the identification and development of DHHC inhibitors is a lack of robust high-throughput screening (HTS) assays. Recently, a yeast-based assay has been reported, in which human PAT acylation of an endogenous PAT substrate is linked to yeast growth via a reporter gene.^17^ While the compounds identified by this assay inhibit substrate *S*-acylation, they may or may not directly inhibit DHHC-PATs and require further validation. Alkyne or isotope labeled palmitoyl-CoA can be used to assess DHHC transferase activity in vitro, but such assays require multiple processing steps and can be difficult to apply in a high-throughput manner.^18^ While the coupled enzyme assay, which detects the release of CoA during the palmitoyl transfer process, is fluorescence-based and therefore suitable for HTS, the indirect readout renders it prone to false positives.^19^ Further, the acylation-coupled lipophilic induction of polarization (acyl-cLIP) assay has been successfully adapted for zDHHC3, zDHHC7, and zDHHC20.^16,20^ In this assay, a fluorophore-labeled peptide is palmitoylated by a DHHC enzyme and inserted into detergent micelles, resulting in a change in fluorescence polarization (FP). However, the dynamic range of this assay is limited, and the peptide substrate requirements for each DHHC isoform are not fully known. Most recently, a FRET-based assay that reports zDHHC2 autoacylation has been used for HTS.^21^ However, the accessibility of bulky non-natural substrate NBD-palmitoyl-CoA is unknown for many DHHCs.

Here, we report the development of palmitoyl transferase probes (PTPs), which are a panel of palmitoyl-CoA mimetic pro-fluorescent probes that report on DHHC activity in vitro. Using the PTPs, we develop a DHHC screening assay with a direct, sensitive, and simple readout of enzyme activity. After validating the assay with three human DHHCs (zDHHC2, zDHHC3, and zDHHC20) and confirming its sensitivity to known DHHC family inhibitors 2BP and CMA, we conduct a pilot screen of a library of 1687 acrylamide-containing molecules against zDHHC20, establishing the suitability of the assay for HTS.

## Results and Discussion

**Figure 1:**
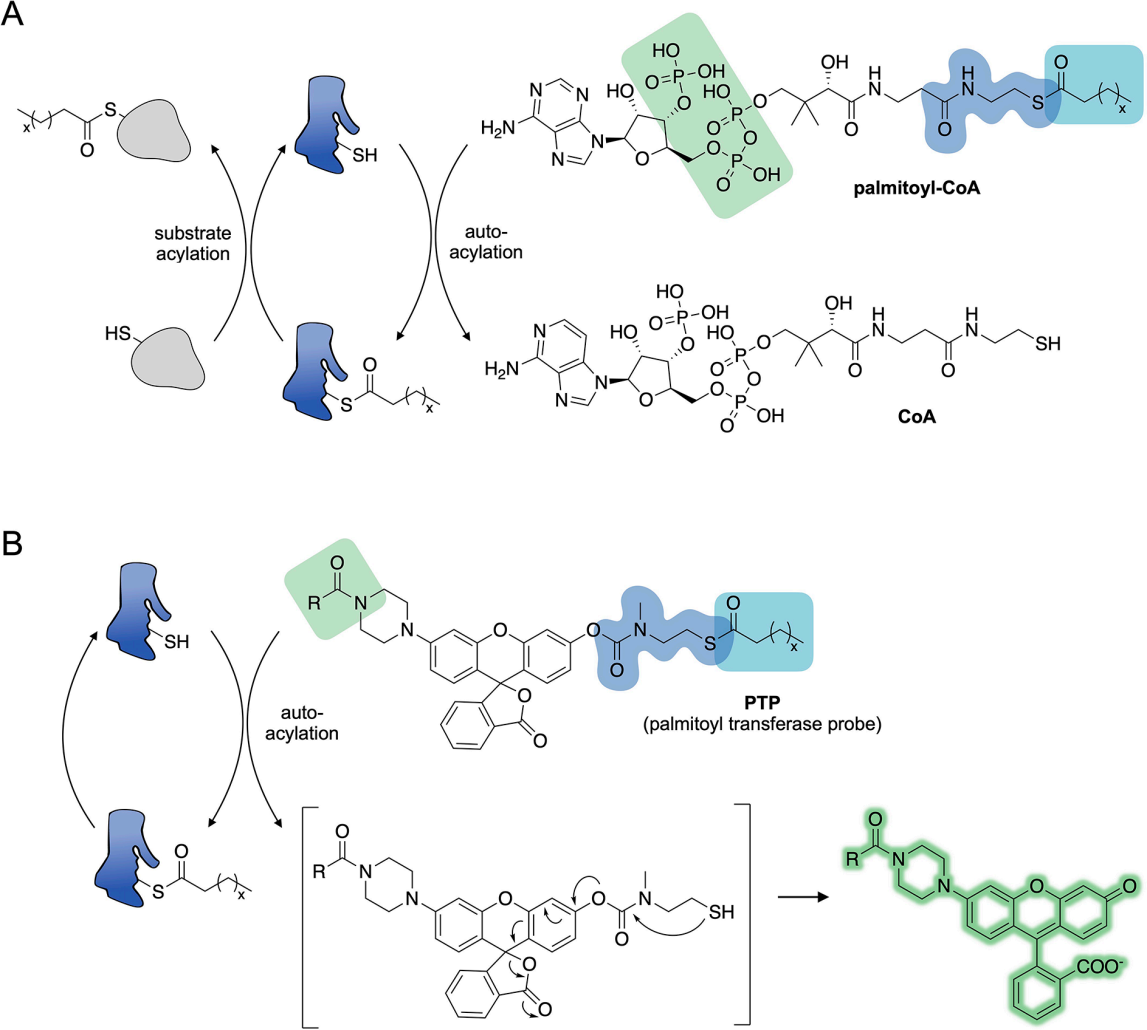
Design of palmitoyl transferase probes (PTPs), CoA substrate-based turn-on probes for DHHC activity. (A) The hypothesized two-step mechanism of DHHC acylation. The enzyme first forms an autoacylated intermediate via nucleophilic attack of the active site cysteine on the acyl-CoA thioester. The DHHC enzyme then transfers the acyl chain to a bound substrate. (B) Design and mechanism of PTPs. An *S*-acylated cysteamine (blue box) is tethered to a pro-fluorescent molecule by a carbamate linker. A DHHC enzyme recognizes the pro-fluorescent molecule as an acyl donor and cleaves the thioester bond of the probe. The free thiol group then cyclizes and cleaves the carbamate linkage to release a fluorescent product (deep green). The isoform specificity and physical properties of the molecule can be tuned by modulating the acyl modification (turquoise box) and functional groups on the piperidine group (light green box).

DHHC catalysis of protein S-acylation is thought to occur via a two-step mechanism.^22^ First, the active site cysteine of the signature DHHC (Asp-His-His-Cys) motif is autoacylated by an acyl-CoA donor, resulting in an acyl:DHHC thioester intermediate. Then, the acyl moiety is transferred from the DHHC cysteine to a protein substrate via a transacylation reaction (Figure 1A). In designing a probe to provide a direct readout of DHHC activity, we sought to exploit the DHHC recognition of the acyl (frequently palmitoyl) CoA donor. We hypothesized that a palmitoyl CoA mimetic fluorescent probe could be recognized by DHHC family proteins, with enzyme processing triggering fluorophore release (Figure 1B). Our laboratory has previously reported *S*-depalmitoylation probes (DPPs), whose deacylation results in thiol-initiated cleavage of a carbamate linker and release of the fluorescent product.^9,23−26^ Intriguingly, we observed that the structure of DPP-5 shares some similarities with palmitoyl CoA, namely, a palmitoyl chain, a cysteamine, and a polar terminal functional group—leading us to posit that this probe could be recognized by DHHC family proteins and serve as a starting scaffold for a fluorescence-based DHHC probe (Figure S1A).^25^ To substantiate this hypothesis, we performed docking in Autodock Vina with a previously reported crystal structure of zDHHC20.^2,27^ Analysis of the lowest-energy conformations revealed that the lipid chain of DPP-5 could be docked into the hydrophobic groove, while its carboxylate could engage with the highly positive adenosine diphosphate (ADP) binding pocket (see Figure S1B in the Supporting Information).

**Figure 2:**
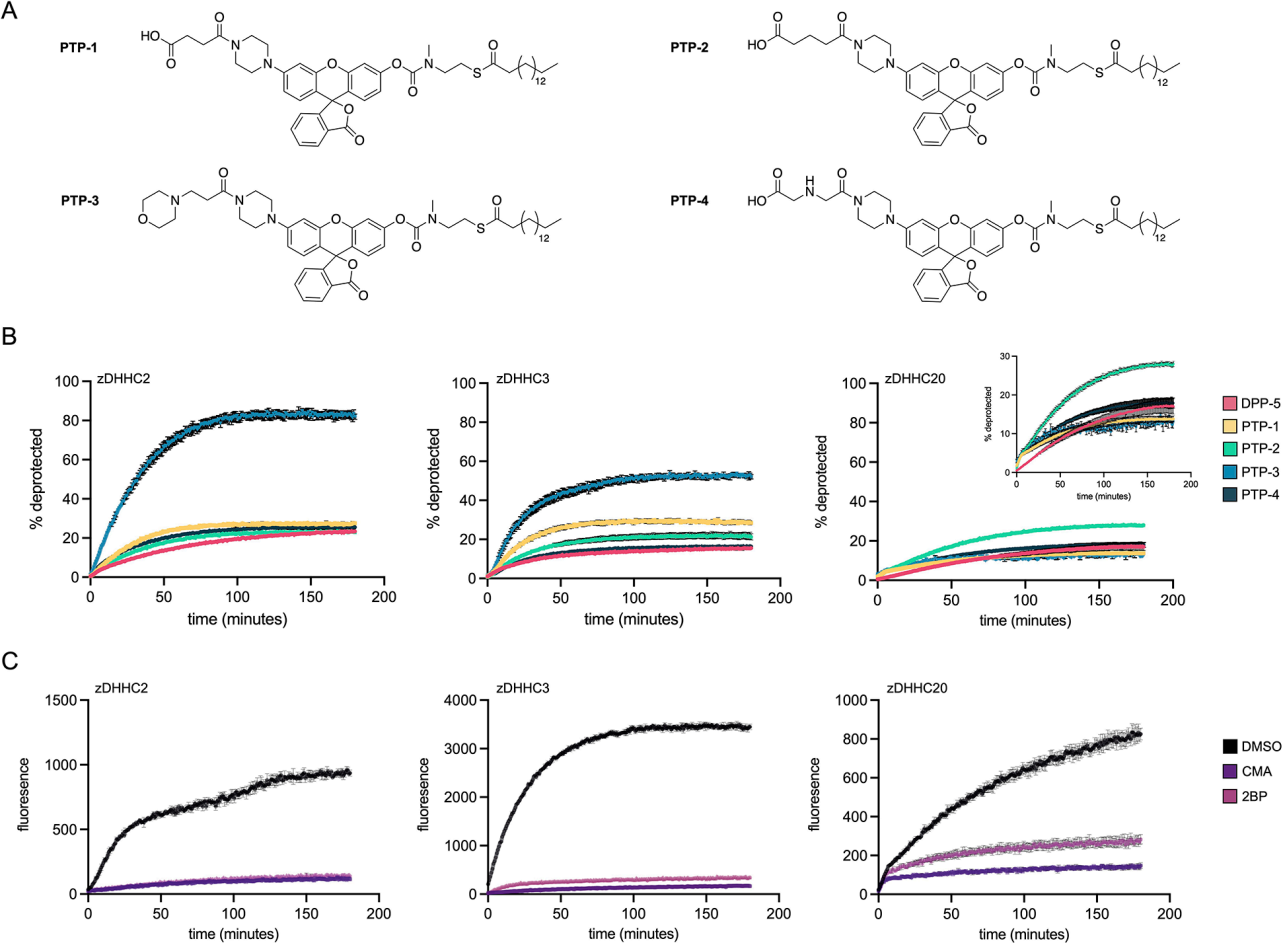
PTPs report DHHC family protein activity. (A) Structures of the family of palmitoyl transferase probes (PTPs) synthesized and tested in this work. All probes feature a palmitoylated cysteamine, with variably modified piperidine groups. (B) Uncaging of PTPs by DHHC family proteins. Each PTP (4 μM) was incubated with purified human zDHHC2, zDHHC3, or zDHHC20 for 3 h. Fluorescent output was monitored over time, normalized to the signal generated by the hydroxylamine (HA)-deprotected probe to give the percent deprotection. Data are presented as the mean ± standard deviation (*n* = 3). (C) Detection of inhibition by PTPs. Preincubation of each zDHHC (zDHHC2, zDHHC3, and zDHHC20) with known DHHC family inhibitors 2BP and CMA (20 μM) abrogated the PTP fluorescent output. Data are presented as the mean ± standard deviation (*n* = 6).

**Scheme 1:**
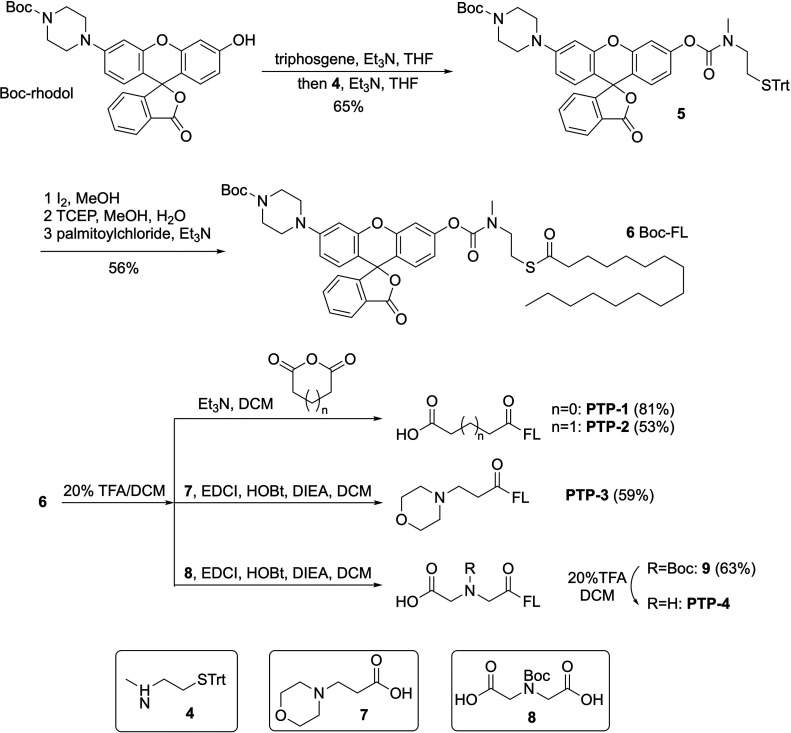
Modular Synthesis of Palmitoyl Transferase Probes (PTPs)

To validate these in silico observations, we then tested the ability of DHHC family proteins to uncage DPP-5 in vitro. Excitingly, incubation of purified human zDHHC2, zDHHC3, and zDHHC20 with DPP-5 resulted in a significant increase in fluorescent signal, confirming that the thioester bond of DPP-5 could be cleaved by DHHC family proteins (see Figure S1A in the Supporting Information). Therefore, we next aimed to optimize this fluorogenic scaffold for interactions with DHHC family proteins and generate a family of palmitoyl transferase probes (PTPs). As an APT substrate, DPP-5 contains a palmitoylated cysteine, which is absent in the palmitoyl CoA substrate of DHHCs (see Figure 1B, as well as Figure S1A). Therefore, we reasoned that removal of the methyl amide from DPP-5 would both minimize steric clashes and result in a better acyl-CoA mimic (**PTP-1**) (see Figure 2A). Docking against zDHHC20 revealed a tightly bound ligand–receptor complex, but also that the probe did not engage a highly conserved key residue of the basic patch, Lys135^2,28^ (Figure S2 in the Supporting Information). In silico screening to maximize this ADP-binding pocket interaction demonstrated that replacement of succinic acid with glutaric acid (**PTP-2**) resulted in further optimized interactions (Figure 2A). Given that different DHHC isoforms might have individualized interactions in the CoA binding region, we also synthesized two additional probes: one with a terminal morpholino group (**PTP-3**) and one with an internal amino group (**PTP-4**) (Figure 2A). Notably, each of these probes shares a native substrate mimetic, a masked fluorophore, and a polar group to facilitate binding. The synthesis of all PTPs proceeded smoothly via a modular synthetic route (Scheme 1). In brief, common precursor **5** was synthesized by triphosgene-mediated carbamate formation between trityl protected cysteamine **4** and **Boc-rhodol**. Following trityl deprotection and lipidation to give PTP precursor **6**, a series of functional groups were installed via acylation to yield **PTP-1–PTP-4**.

With this small panel of probes in hand, we first screened them against zDHHC20 to identify the best substrate, i.e., the most uncaged molecule, for this protein. While all probes gave significant fluorescent signal, **PTP-2** emerged as the probe most deprotected by zDHHC20, paralleling the docking experiments (see Figure 2B, as well as Figures S1B and S2–S5). Moreover, incubation with the DHHA mutant of zDHHC20, which is a variant lacking a key catalytic cysteine, resulted in an 80% decrease in fluorescent signal (see Figure S6A in the Supporting Information). We next measured the enzyme kinetics to elucidate the affinity of the zDHHC20**/PTP-2** interaction. We determined the values of *K*_m_ and *k*_cat_ to be 24.3 μM and 0.160 s^–1^, respectively, while the calculated *k*_cat_/*K*_m_ value was 6.58 × 10^3^ M^–1^ s^–1^ (see Figures S6B and S6C in the Supporting Information). These kinetic parameters are comparable to those of the natural substrate palmitoyl-CoA, suggesting that **PTP-2** can indeed behave as an acyl-CoA mimic. Finally, to establish the adaptability of this assay for other DHHC family proteins, we also screened the probes against zDHHC2 and 3. Interestingly, we observed here that **PTP-3**, the morpholino compound, was the best substrate for these two DHHCs (Figure 2B). Together, these data confirm the ability of the PTP fluorogenic probe family to report on the activity of DHHC family proteins in vitro.

We next sought to establish the suitability of this assay for a high-throughput screen. The Z′-factor is a statistical characteristic used to evaluate the quality of an assay, with a Z′-factor of >0.5, indicating congruence with an HTS.^29^ Here, evaluation of the Z′-factor during the linear reaction rate time (*t* = 30 min) afforded a value of 0.77 for zDHHC20. This Z′-factor compares favorably with other published assays, including DHHC-acyl-cLIP (Z′ = 0.553), and reflects the large dynamic range (∼1000 RFU) of the assay. A similarly robust Z′-factor was observed for zDHHC2 and zDHHC3 (*t* = 30 min, 0.78 and 0.83, respectively). We then confirmed the ability of this assay to detect DHHC inhibition using known inhibitors. ZDHHC2, zDHHC3, and zDHHC20 were incubated with 20 μM of either 2BP or CMA and the fluorescent readout recorded. Here, we observed that both CMA and 2BP were able to inhibit zDHHC2, zDHHC3, and zDHHC20 (Figure 2C). Specifically, CMA and 2BP were found to have IC_50_ values of 0.463 ± 0.07 μM and 2.020 ± 0.29 μM (see Figures S7A and S7B in the Supporting Information), respectively, comparable to those reported by the zDHHC20 acyl-cLIP assay (1.35 ± 0.26 μM and 5.33 ± 0.77 μM). These results establish the utility of the PTP-based fluorescence assay for HTS.

**Figure 3:**
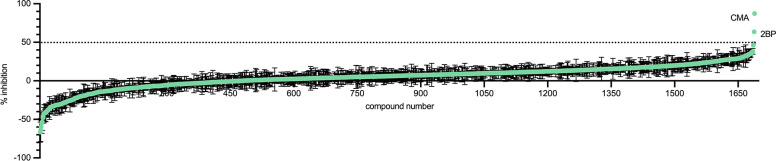
Screen of an acrylamide library against zDHHC20. A 1687-member acrylamide library was screened against zDHHC20 using **PTP-2**, with CMA and 2BP included as positive controls. Data are presented as the mean ± standard deviation (*n* = 2). Hits were defined as inhibiting enzyme activity by at least 50% at 25 μM (dotted line).

Having validated the compatibility of the assay for HTS, we next conducted a pilot screen. As our previous work has verified the ability of acrylamide-containing molecules to inhibit DHHC family proteins, we screened a library of 1687 acrylamide-containing compounds in a 384-well plate format at a fixed concentration of 25 μM, with CMA and 2BP as positive controls (Figure 3A). With a threshold set for inhibition of 50% or more at 25 μM, we found that none of the molecules in this library exhibited inhibitory activity, even while CMA and 2BP both displayed over 70% inhibition of zDHHC20. These same results were observed when screening a subset of the library using the FP-based acyl-cLIP assay, wherein the library compounds demonstrated ≤20% inhibition, while CMA and 2BP abrogated zDHHC20 activity (see Figure S8 in the Supporting Information). These data—detection of inhibiting and noninhibiting compounds—confirm the suitability of this assay as a high-throughput method to identify DHHC inhibitors.

The increasing awareness of the importance of DHHC activity in both health and disease underscores the need for chemical inhibitors to probe the biology and therapeutic potential of these targets. However, the development of inhibitors is hindered by the limitations of current biochemical assays for DHHC activity. In this Letter, we introduce a fluorescence-based assay for DHHC activity based on CoA substrate mimetics—a rare small molecule-based assay for a PTM writer protein. Using in silico modeling as a guide, we rationally designed a panel of pro-fluorescent PTPs that capitalize on DHHC recognition of palmitoyl-CoA during the autoacylation step of its catalytic cycle, uncaging the probe and providing a turn-on fluorescent readout of DHHC activity. The flexible and modular synthesis of the PTPs suggests that the PTP library could be easily expanded to other DHHC family members and even adapted to other transferases, such as *N*-myristoyltransferase family proteins (NMTs). We demonstrate that this assay is amenable to high throughput screening of DHHC inhibitors based on the fluorescence readout, reagent cost and quantity, and excellent Z′-factors for three DHHC protein family members (0.77–0.84). However, although PTP screening demands less-purified DHHC protein than published assays, it is still limited by the fact that only a handful of these transmembrane proteins have been purified.

In summary, we have established PTPs as pro-fluorescent substrate mimetics for DHHC family proteins that provide a readout of DHHC activity in vitro. As a proof-of-concept for HTS, we used the PTP-based fluorescence assay to screen 1687 acrylamide-based molecules against zDHHC20 in a 384-well plate format. While no potent, nonlipidic compounds were identified in this pilot screen, we anticipate that a larger HTS has the potential to identify more druglike, and perhaps isoform-selective, DHHC inhibitors.

## Supporting Information Available

Supplementary figures and schemes, materials and methods, detailed HTS protocol, synthetic methods and characterization (PDF)
