# Supplementary material for: A High-Throughput Fluorescent Turn-On Assay for Inhibitors of DHHC Family Proteins
Source: ACS Chem Biol. 2022 Jul 11;17(8):2018–23. doi: 10.1021/acschembio.2c00193 (PMC9391280; doi:10.1021/acschembio.2c00193)
Supplement: Supplementary file 1 — cb2c00193_si_001.pdf [file cb2c00193_si_001.pdf]

SUPPORTING INFORMATION

A high-throughput fluorescent turn-on assay for inhibitors of DHHC family proteins

Tian Qiu,[1#] Saara-Anne Azizi,[1,2#] Noah Brookes,[1] Tong Lan[1] and Bryan C. Dickinson[1*]

[1]Department of Chemistry, The University of Chicago, Chicago, Illinois 60637, United States
[2]Medical Scientist Training Program, Pritzker School of Medicine, The University of Chicago, Chicago, Illinois 60637, United States

*Dickinson@uchicago.edu

SUPPLEMENTARY FIGURES AND SCHEMES…………………………………….. S2-S11

MATERIALS AND METHODS…………………………………………………………..S12-S15

DETAILED HTS PROTOCOL FOR USING PTPs TO SCREEN DHHCs…………..S16-S17

SYNTHETIC METHODS AND CHARACTERIZATION……………………………… S18-S44

REFERENCES……………………………………………………………………………… S45

SUPPLEMENTARY FIGURES AND SCHEMES

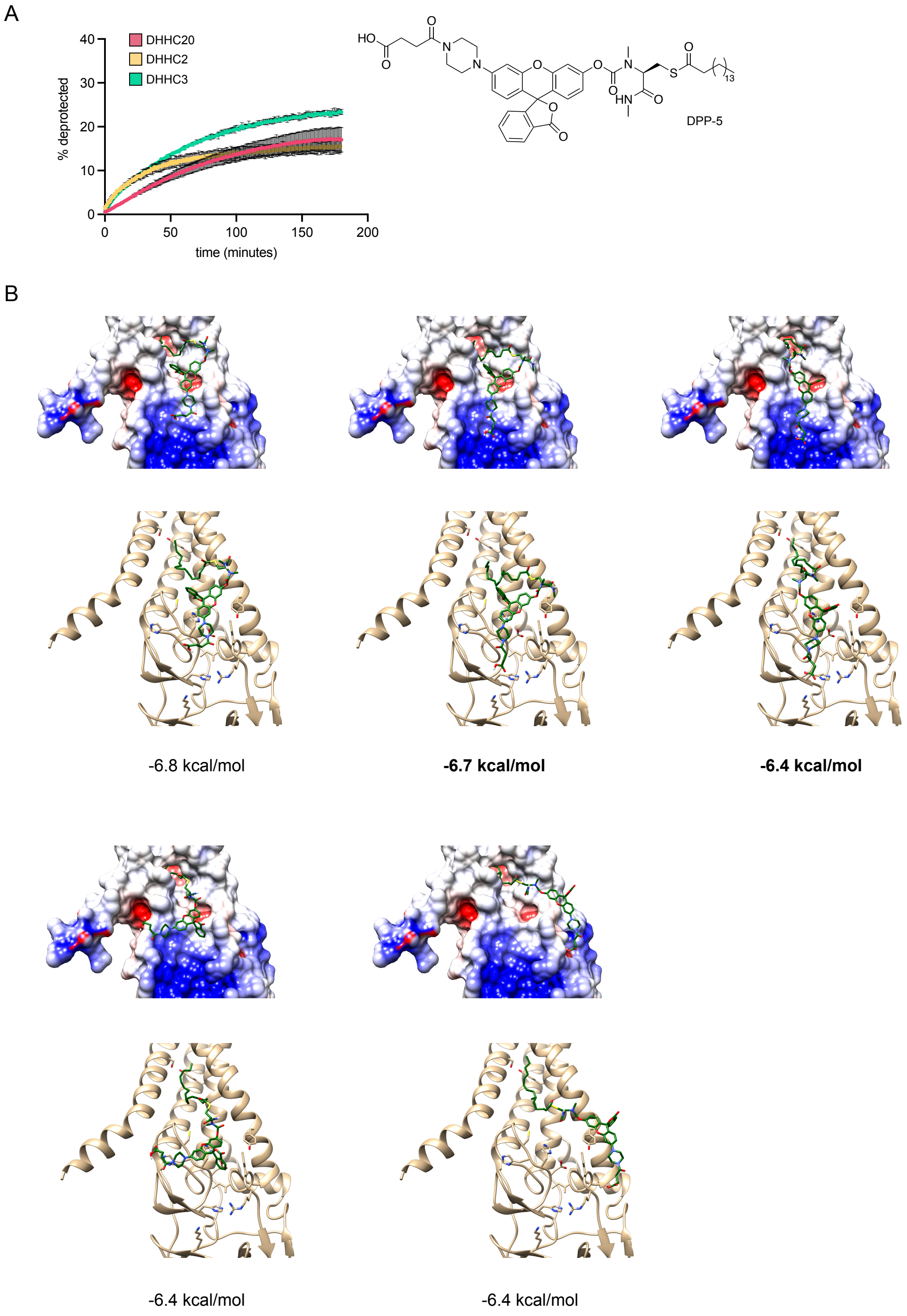

Figure S1. *In vitro* and *in silico* validation of the DPP-5/DHHC interaction. (A) DPP-5 can be uncaged by DHHC family interactions. Purified zDHHC2, 3, and 20 were incubated with DPP-5 (4 μM), and the resulting fluorescence signal was normalized to the signal generated by the

hydroxylamine (HA)-deprotected probe to give the percent deprotection. Data are presented as the mean ± standard deviation (n = 3). (B) Docking studies of DPP-5 (green) with zDHHC20 (PDB: 6BML). Surface colored by electrostatic potential (top) and cartoon (bottom) views of the representative low energy conformations. Electrostatic surface potential was generated using an Adaptive Poisson-Boltzmann Solver (APBS) server with default settings. Positively (blue) and negatively (red) charged surfaces are displayed at the contour levels of +5 and–5 kBT/e, respectively, where kB is the Boltzmann constant, T is temperature, and e is the charge on an electron. The predicted ∆G values of binding are labeled below each pose, and the ∆G values of poses which have interactions with ADP-binding patch are highlighted boldly. Selected hydrogen bond interactions are shown as dashed yellow lines. The side chain of key residues is highlighted.

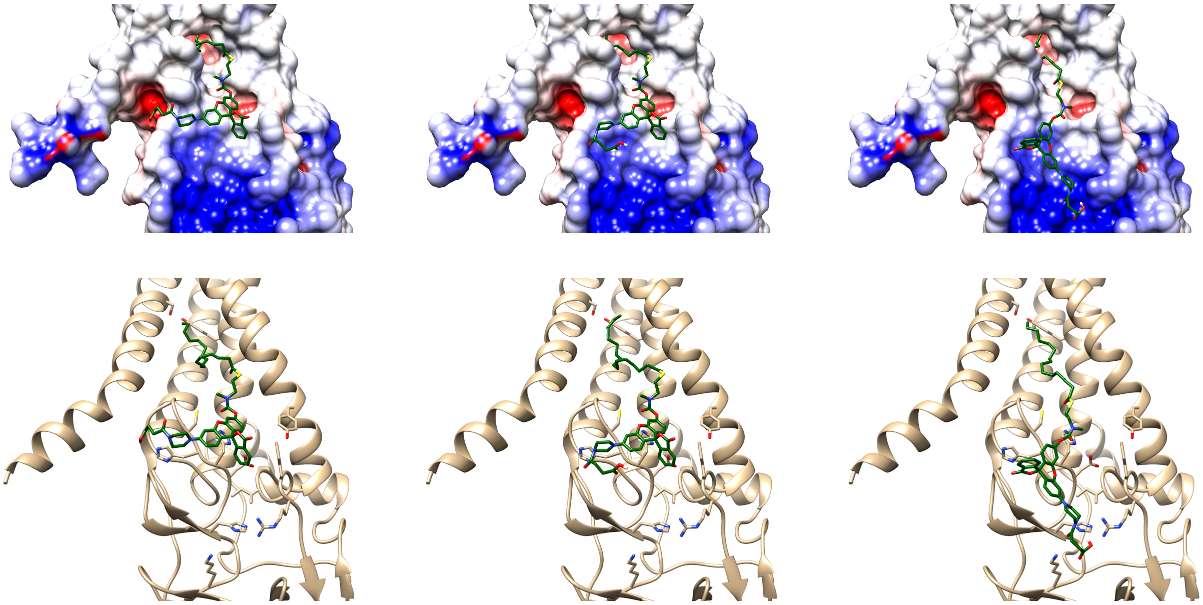

-8.7 kcal/mol | -8.4 kcal/mol | **-7.7 kcal/mol**

Figure S2. Docking studies of PTP-1 (green) with zDHHC20 (PDB: 6BML). Surface colored by electrostatic potential (top) and cartoon (bottom) views of the representative low energy conformations. The predicted ΔG values of binding are labeled below each pose, and the ΔG values of poses which have interactions with ADP-binding patch are highlighted boldly.

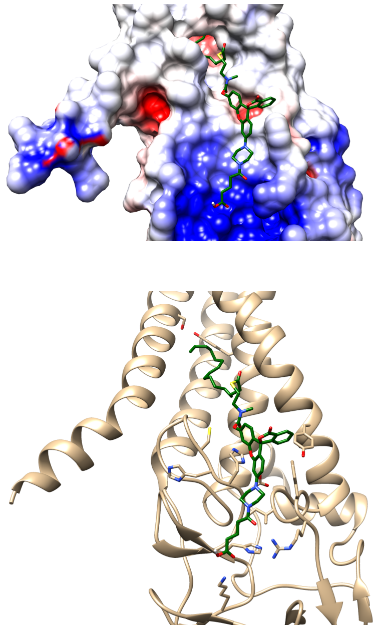

-8.0 kcal/mol | **-7.4 kcal/mol** | -7.2 kcal/mol

Figure S3. Docking studies of PTP-2 (green) with zDHHC20 (PDB: 6BML). Surface colored by electrostatic potential (top) and cartoon (bottom) views of the representative low energy conformations. The side chain of key residues is highlighted. The predicted ΔG values of binding are labeled below each pose, and the ΔG values of poses which have interactions with ADP-binding patch are highlighted boldly.

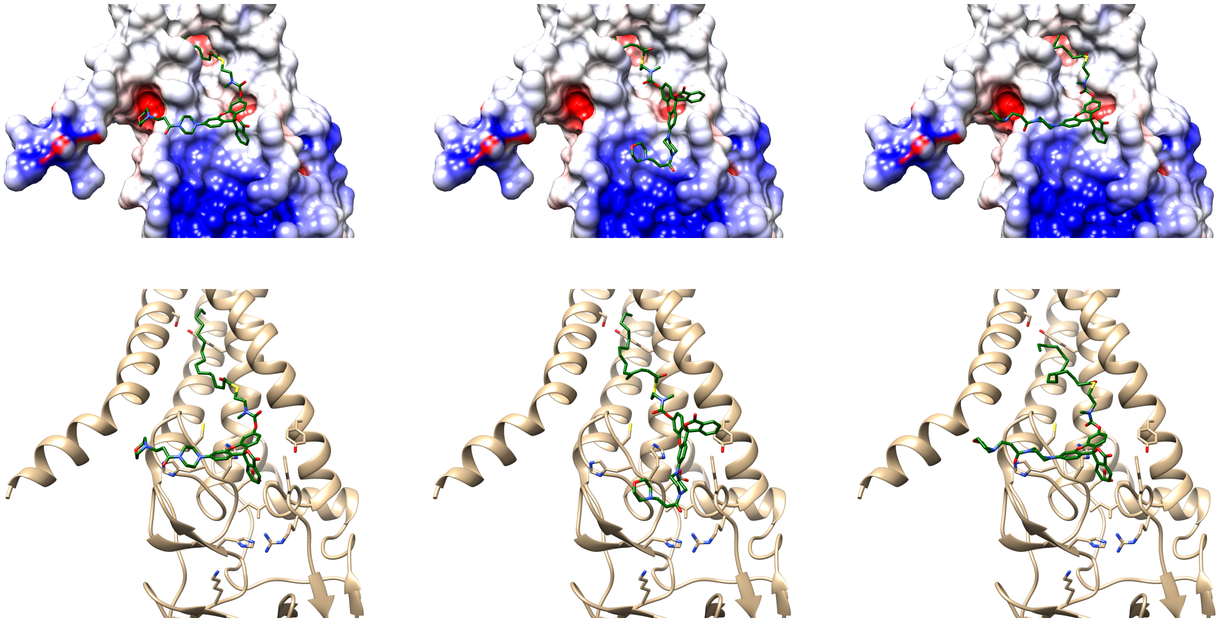

-8.7 kcal/mol -8.4 kcal/mol -8.4 kcal/mol

Figure S4. Docking studies of PTP-3 (green) with zDHHC20 (PDB: 6BML). Surface colored by electrostatic potential (top) and cartoon (bottom) views of the representative low energy conformations. The side chain of key residues is highlighted. The predicted ΔG values of binding are labeled below each pose, and the ΔG values of poses which have interactions with ADP-binding patch are highlighted boldly.

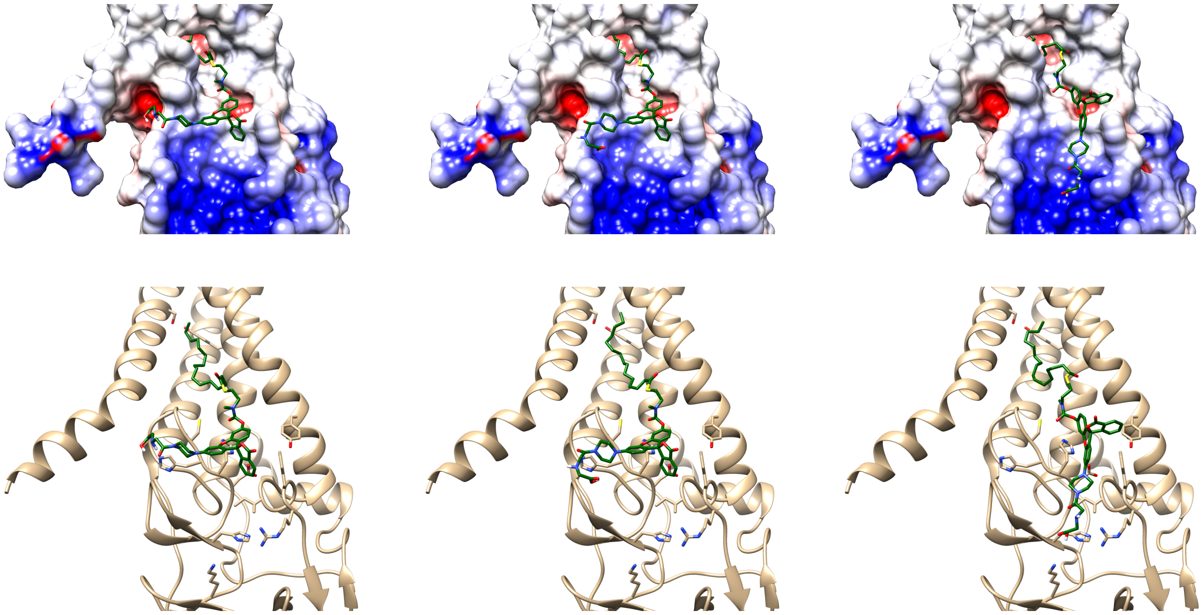

-8.4 kcal/mol -8.1 kcal/mol **-7.7 kcal/mol**

Figure S5. Docking studies of PTP-4 (green) with zDHHC20 (PDB: 6BML). Surface colored by electrostatic potential (top) and cartoon (bottom) views of the representative low energy conformations. The side chain of key residues is highlighted. The predicted ΔG values of binding are labeled below each pose, and the ΔG values of poses which have interactions with ADP-binding patch are highlighted boldly.

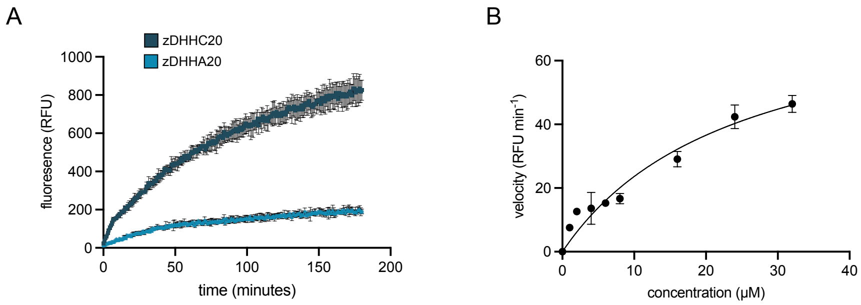

C

	PTP-2	palmitoyl-CoA
K_m	24.3 μM	0.58 μM
k_{cat}	0.16 s^{-1}	0.275 s^{-1}
k_{cat}/K_m	6.58 x 10^3 M^{-1} s^{-1}	4.74 x 10^5 M^{-1} s^{-1}

Figure S6. Validation and characterization of PTP-2. (A) PTP-2 (4 μM) was incubated with either WT zDHHC20 or its inactive mutant (DHHA) and the fluorescent output (PTP-2 uncaging) was monitored over time. Data are presented as the mean ± standard deviation (n=3). (B) zDHHC20 was incubated with PTP-2 at a 1, 2, 4, 6, 8, 16, 24, and 32 μM, and the initial velocity (RFU/min) was plotted against the stated concentrations to give the K_m and K_{cat}. (C) Tabulation of kinetic parameters for PTP-2 and palmitoyl CoA with zDHHC20.[1]

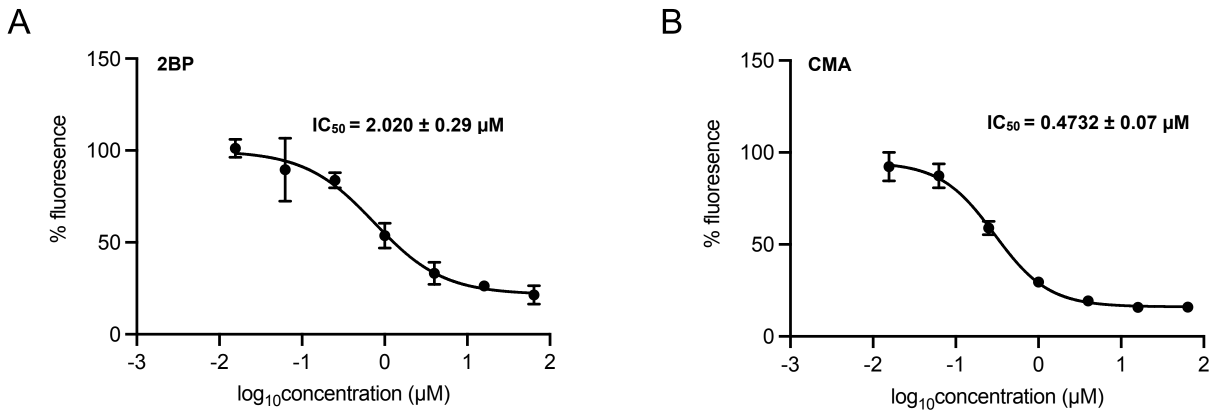

Figure S7. Validation of the fluorescence assay using known inhibitors. Dose-response curves of 2BP (A) and CMA (B) inhibition of zDHHC20 with a pre-incubation time of 30 min, measured by the fluorescent assay with PTP-2. The IC50 values were calculated from a four-parameter dose-response curve, and the data represented mean ± standard deviation (n = 3).

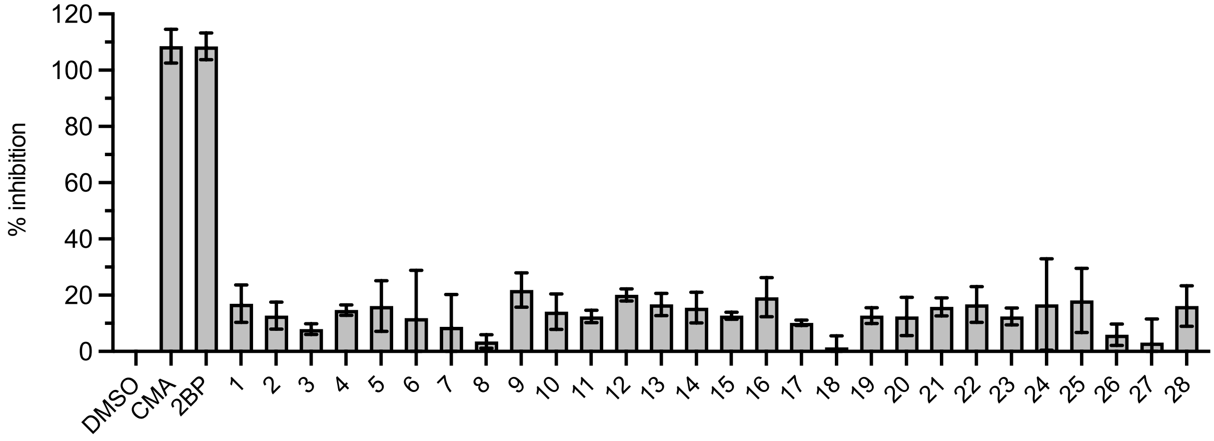

Figure S8. Secondary validation of screening of a subset of compounds (Compounds 1-20 are first 20 compounds that have the highest inhibition, compounds 21-28 are 8 compounds that have nearly no inhibition) using a fluorescence polarization assay. Fluorescence polarization (FP) screening of selected hit compounds against zDHHC20, with activity normalized to DMSO. Data are presented as the mean ± standard deviation (n=3).

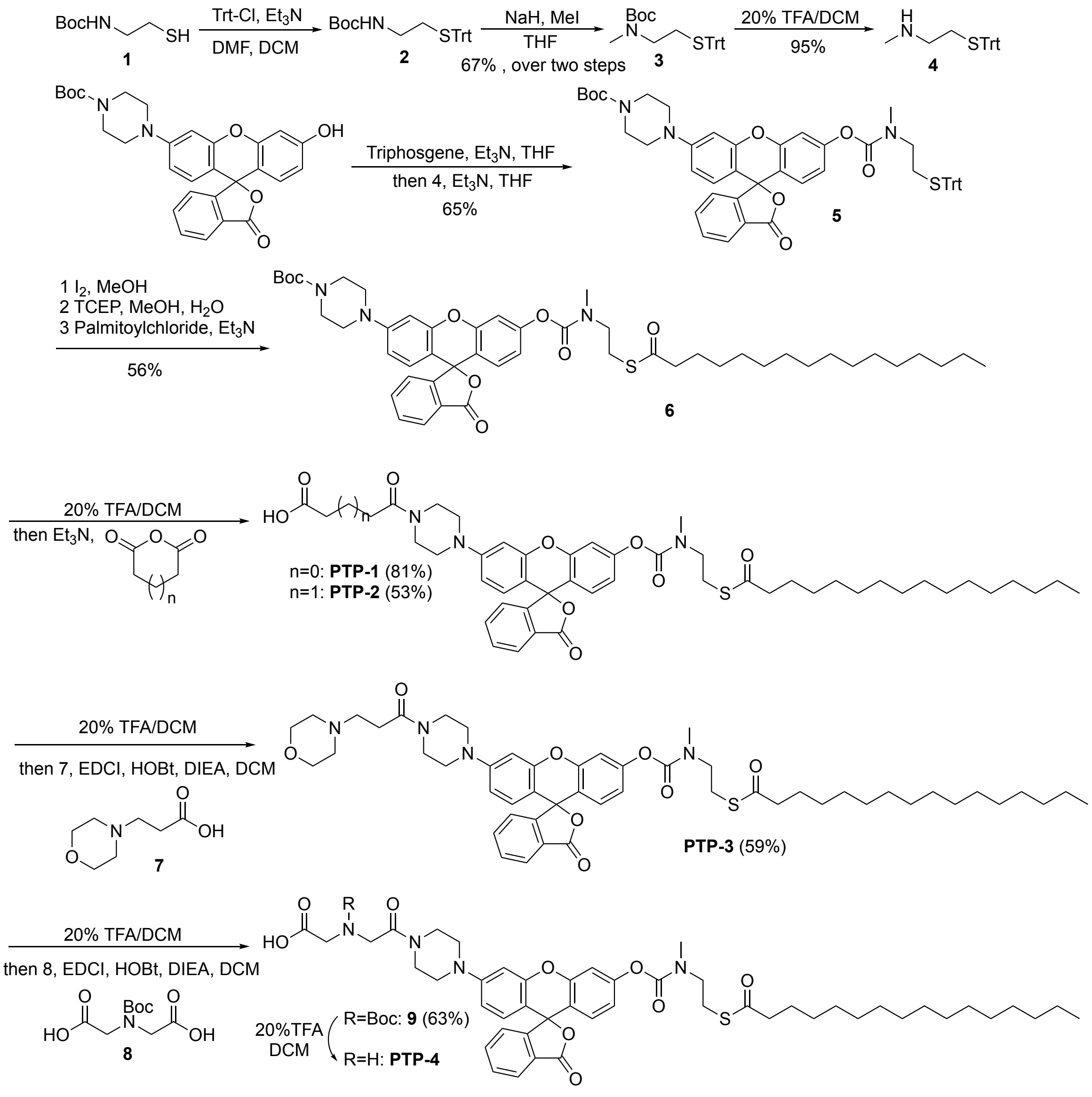

Scheme S1. Synthesis of PTPs. Synthetic scheme for PTP-1 though PTP-4, highlighting the modularity of the synthesis and thus its adaptability for additional DHHC isoforms or other acyl transferase proteins.

MATERIALS AND METHODS

General materials

For biochemical and biological experiments, TCEP (UBPBio), n-Dodecyl-β-D-maltoside (DDM) (Cayman chemical) and 2-bromopalmitate (Sigma) were purchased from the sources given in parentheses. DPP-5, CMA, and RSK6 were synthesized as previously reported.[2] Docking was performed with the AutoDock Vina (v. 1.2.0).[3] Preparation of the receptor and the ligands was done with the MGL Autodock Tools package (ADT). The human zDHHC20 structure (PDB: 6BML) without 2BP was used as the receptor. The allowed conformational space, the "docking box," was centered on the 2BP binding pocket as defined by Rana et al. (PDB: 6BML). For each ligand, 100000 models were generated, and 20 protein-ligand complexes with lowest energy were selected for analysis. The results were analyzed with Chimera.[4]

Plasmid cloning

All newly cloned plasmids were constructed by Gibson assembly from PCR products generated using Q5 Hot Start DNA Polymerase (New England Biolabs). The human DHHC2/3/20 in pCE-puro backbone was a gift from S. Bamji (University of British Columbia). Plasmid pcDNA 6.1 eGFP-GobX was a gift from M. Machner at NIH, and GobX was cloned into pET-30A containing a His6 tag at N-terminal for E. coli expression as described previously.[2] All customized primers were synthesized by Integrated DNA Technologies (IDT). All of the DHHC library plasmids and newly constructed plasmids used in this work were sequence-verified at the University of Chicago Comprehensive Cancer Center DNA Sequencing and Genotyping Facility and are available on request.

Purification of human zDHHC2, 3, and 20

Expression, Hek293T cells:

HEK293T cells (~3 x 10^6 cells per dish) were plated in a 15 cm dish (Corning). After 24 hours, at ~70-80% confluency, cells were transfected with 33 µg of pCE-puro myc-His6-human zDHHC20 isoform 3 using PEI. Two days post transfection, cells were harvested and washed twice with DPBS. Protein obtained from HEK293T cells was used from for characterization and validation of the assay (probe screening, DHHC20/PTP-2 interactions.

Expression, Sf9 cells:

Recombinant baculovirus encoding myc-His_6-human zDHHC20 was generated by cloning myc-His_6-human zDHHC20 into a pFastBac1 vector and obtained following transfection and two rounds of amplification in Sf9 cells (Novagen), as previously reported.[5] Then, 200 mL of Sf9 insect cells (2.5 x 10^6 cells/mL) were infected with the baculovirus encoding cloning human zDHHC20.

After incubation at 28 °C for 48 h, infected cells were harvested by centrifugation at 3000x*g* and then washed with DPBS and flash frozen until purification. Protein purified from Sf9 cells was used for HTS screening;

Purification:

Cells were lysed with the lysis buffer (50 mM Tris, 150 mM NaCl, 1 mM EDTA, 10% glycerol, 1 mM TCEP, pH 7.4 with 20 mM DDM and a protease inhibitor cocktail) and rotated end-over-end at 4 ˚C for 1 hour. After centrifugation at 12,000x*g* at 4℃ for 40 min, cell debris was discarded, and the cell lysate was diluted twice with DDM-free lysis buffer before incubation with Takara His60 Ni Superflow Resin (500 μL for a 15 cm dish of HEK293T cells and 3 mL for a 200 mL culture of Sf9 cells) via end-over-end rotation at 4℃ for 1 hour. The Ni resin was then washed with equilibrium buffer (10 mM imidazole, 50 mM Tris, 1 M NaCl, 20% glycerol, 10 mM TCEP, pH 7.5) and spun down at 4,000 rpm in 4℃ for 5 min. After three wash cycles, the His-tagged proteins were eluted via incubation (3x) with a column volume of elution buffer (300 mM imidazole, 50 mM Tris, 1 M NaCl, 20% glycerol, 10 mM TCEP, pH 7.5). The purity of the eluted fractions was assessed by SDS-PAGE gel electrophoresis, and the presence of each zDHHC protein was confirmed via anti-myc Western blotting. The fractions with zDHHC proteins were combined and concentrated as needed using the Amicon Ultra-4 Centrifugal Filter Unit, 10 kDa. The concentration of zDHHC 20 was determined initially by Bradford assay (Bio-Rad) and for subsequent batches, by fluorescence polarization (FP) assay. The concentrations of zDHHC2 and 3 were determined by Coomassie Blue. Purified protein was stored at -20℃ (for immediate use) or -80℃ (for long-term storage).

In vitro **fluorescence polarization (FP) assay**

The FP assay was adopted from a previously method[2] and used to validate selected compound inhibition. Here, a 48 μl protocol with 1 hour inhibitor preincubation was used, and all inhibitors were tested at 25 μM concentration. Measurements were recorded on Synergy Neo2 Hybrid Multi-Mode Reader (BioTek Instruments, Inc.) with Dual FP Green filter cube. Fluorescence polarization (λ_{ex} = 485/20 nm, λ_{em} = 528/20 nm, gain = 35, top reading with height 8.0 mm, and filter switching method) was measured at 1-min time intervals for 1 hour at 37℃. Assay data were exported in Microsoft Excel. The assay was performed in kinetics mode, and the difference taken between average values at the end (t = 114-120 min) and the beginning (t = 0-6 min) was used to determine the enzyme activity.

In vitro **fluorescence-based palmitoyl transferase probe (PTP) assay**

PTP screening against DHHC2/3/20

To balance sensitivity, replicability, and resources (DHHC enzyme), 20 μL was determined to be the lowest tractable volume for all studies. For a 20 μL protocol, 1.5 μL of purified human zDHHC2/3/20 enzyme (with the concentration across batches normalized to FP = 40 at 1 hour) in protein storage buffer and 8.5 μL of PTP reaction buffer (PBS, with 2 μM TCEP, pH=7.4) were added to a 384-well optical bottom plate (ThermoFisher). PTP reaction buffer with protein storage buffer (total volume 10uL) was used as a blank. Then, 10 μL of 8 μM of **PTPs 1-4** in PTP reaction buffer was added to initiate the reaction, resulting in a final concentration of 4 μM PTP. Fluorescence measurements (λ_{ex} = 485/20 nm, λ_{em} = 528/20 nm, gain=80, top reading with height 8.0 mm) were recorded on Synergy Neo2 Hybrid Multi-Mode Reader (BioTek Instruments, Inc.) in kinetics mode at 10-min time intervals for 2 hours at 37℃. Assay data were exported to Microsoft Excel and analyzed using Prism 9 (GraphPad).

Known inhibitor validation

For a 20 μL protocol, 1.5 μL of purified human zDHHC2, 3, or 20 in protein storage buffer and 6.5 μL of PTP reaction buffer (PBS, with 2 μM TCEP, pH=7.4) were added to a 384-well optical bottom plate (ThermoFisher). Then, 2 μL of each 5x inhibitor stock (CMA or 2BP) in PTP reaction buffer was added, resulting in a 10 uL total volume mixture with 20 μM inhibitor. The resulting reactions were incubated in 37℃ for 1 hour. Before the addition of the PTP solution, 8 μL of 10 μM PTP (**PTP-2** or **PTP-3**) in PTP reaction buffer was mixed with 2 μL of 5x inhibitor stock in PTP reaction buffer. This solution was added to the 384-well plate to initiate the reaction, resulting in a final PTP concentration of 4 μM while also maintaining the inhibitor concentration. To determine IC_{50} values, the final concentrations of each inhibitor (2BP and CMA) were 0, 0.0156, 0.0625, 0.25, 1, 4, 16, and 64 μM. Fluorescence measurements (λ_{ex} = 485/20 nm, λ_{em} = 528/20 nm, gain=80, top reading with height 8.0 mm) were recorded on Synergy Neo2 Hybrid Multi-Mode Reader (BioTek Instruments, Inc.) in kinetics model with 45-sec intervals (inhibitor validation) and 2-min time intervals (IC_{50}) for 2 hours at 37℃. Assay data were exported to Microsoft Excel and analyzed using a four-parameter dose-response curve in Prism 9 (GraphPad).

PTP kinetic parameter measurement

1.5 μL of purified human zDHHC20 enzyme in protein storage buffer and 8.5 μL of PTP reaction buffer (PBS, with 2 μM TCEP, pH=7.4) were added to a 384-well optical bottom plate (ThermoFisher). 10 μL of **PTP-2** at a range of concentrations in PTP reaction buffer was added to initiate the reaction, resulting in 1, 2, 4, 6, 8, 16, 24, and 32 μM **PTP-2** in the final reaction. Fluorescence measurements (λ_{ex} = 485/20 nm, λ_{em} = 528/20 nm, gain=80, top reading with height 8.0 mm) were recorded on Synergy Neo2 Hybrid Multi-Mode Reader (BioTek Instruments, Inc.) in kinetics model with 45-second intervals for 2 hours at 37℃. Assay data were exported in

Microsoft Excel, and the initial velocity was calculated from linear regression analysis of the first 8 data points.

High-throughput screening

The chemical library (1056890-Y25) was purchased from ENAMINE, and all compounds were screened at 25 μM concentration. The step-by-step protocol can be found below.

Protocol for PTP-based HTS screen

Notes:

1. The concentration of purified protein can differ among batches; we normalized the concentration to FP=40 (60 min.) for zDHHC20.
2. We suggest including a known inhibitor, such as 2BP or CMA, as a positive control.
3. The recommended 384-well plate layouts for both automated and manual HTS screens are shown at the end of each protocol.

Reagent Preparation

- PTP reaction buffer (1x PBS, 2 µM TCEP, pH=7.4)
- PTP stock solution: 5 mM in DMSO
- 5 mM stocks of library compounds and positive control

Manually HTS screening

1. Determine plate layout (see Plates 1&2)
2. Prepare Master Mix I (MMI): PTP reaction buffer and purified zDHHC enzyme
   N samples, two replicates each
   a. Measure enzyme (1.5 µL/reaction): 1.5*(2*(N+8)) µL
   b. Measure buffer (6.5 uL/reaction): 6.5*(2*(N+8)) µL
   c. Add the calculated amount of enzyme (a) into the calculated amount of buffer (b), mix well, and store on ice.
3. Dilute inhibitors into reaction buffer (note: we found a 96 well plate is superior for solution preparation).
   a. Determine the 96 well plate layout (see Plate 3)
   b. Add 117 µL of PTP reaction buffer to each well of 96-well plate A.
   c. Add 3 µL 5 mM stock solution of inhibitor/control into the appropriate well to give 125 µM solutions, mixing well; for the negative control, DMSO is added instead.

4. Add 8 µL MMI into the experimental wells of the 384-well plate. For the PTP-only blank well, add 8 µL PTP reaction buffer instead.

5. Using a 10 µL multichannel pipette, transfer 2 µL inhibitor solution from 96 well plate A to the 384-well plate, mixing once. Place the 384-well plate at 37 ˚C for 30 min. of preincubation.

6. Prepare Master Mix II (MMII): PTP and inhibitor solution
   a. Dilute 6 µL of 5 mM PTP stock solution into 3 mL PTP reaction buffer to give a 10 µM PTP solution. Add 2 µL DMSO into 1 mL PTP reaction buffer to give a DMSO control solution.
   b. Add 30 µL of the 10 µM PTP solution to each well in 96 well plate B, using 30 µL DMSO control solution for the well with the enzyme-only blank.
   c. Add 7.5 µL of inhibitor solution or DMSO to corresponding wells to make MMIII MMII contains 8 µM PTP and 25 µM inhibitor.

7. Using 10 µL multichannel pipette, transfer 10 µL of MMII from 96 well plate B to the 384-well plate and mix once. Place plate into plater reader (Synergy Neo2 Hybrid Multi-Mode Reader, BioTek Instruments) immediately.

8. Record fluorescence measurements (λ_{ex} = 485/20 nm, λ_{em} = 528/20 nm, gain=80, from the top with height 8.0 mm) in kinetics model with 10-min time intervals for 2 hours at 37 ˚C and export data to Microsoft Excel.

Plate 1: 384-well plate, screening layout – manual

- A1/A24/P1/P24: positive control
- C1/C24/N1/N24: DMSO
- E1/E24/L1/L24: no enzyme
- G1/G24/J1/J24: no PTP
- A2-A23/C2-C23/E2-E23/G2-G23/I2-I23/K2-K23/M2-M23/O2-O23: screening region
- Compounds screened (considering positive control, DMSO, and blank wells):
  2 Replicates: 86 compounds/plate
  3 Replicates: 58 compounds/plate

Note that we observed manual multichannel pipetting was more reliable than electronic

Plate 2: 384-well plate, screening layout – automatic

- A1/A24/P1/P24: positive control
- A2/A23/P2/P23: DMSO
- A3/A22/P3/P22: no enzyme
- A4/A21/P4/P21: no PTP
- B1-O24: screening region
- Compounds screened (considering positive control, DMSO, and blank wells):
  2 Replicates: 166 compounds/plate
  3 Replicates: 110 compounds/plate

Plate 3: 96-well plate A, dilution of library compounds

In a 96 well plate, A1-H11 contain inhibitors to be tested. The well labels correspond to the inhibitor's final position in the 384-well plate.

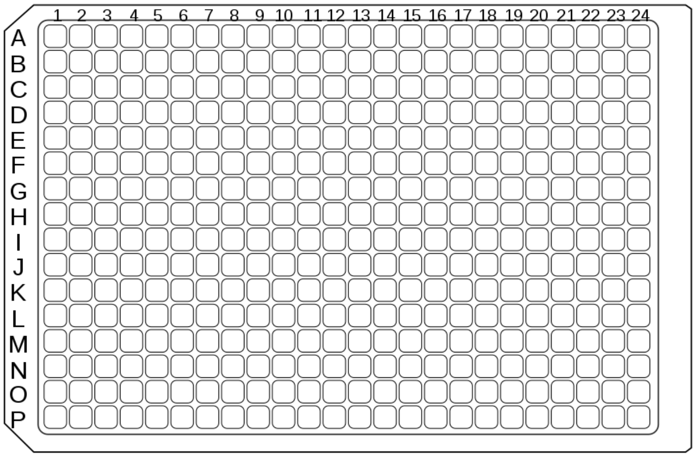

Plates 1&2

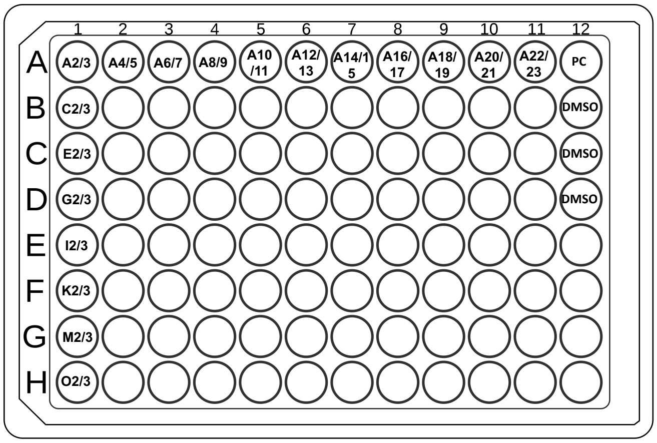

Plate 3

SYNTHETIC METHODS AND CHARACTERIZATION

General materials

For chemical synthesis, reagents and solvents were purchased from commercial sources and used without further purification. Silica gel P60 (SiliCycle, 40–63 μM, 230–400 mesh) was used for column chromatography. Analytical thin-layer chromatography was performed using SiliCycle 60 F254 silica gel (pre-coated sheets, 0.25 mm thick) with detection at 214 nm. Low-resolution-mass spectral analysis and liquid chromatography analysis were carried out on an Advion Expression-L mass spectrometer (Ithaca, NY) with electron spray ionization (ESI) in the positive mode coupled to an Agilent 1220 Infinity LC System with an Agilent Poroshell 120 column (Santa Clara, CA). Automated flash column chromatography purification was carried out on a Biotage system Isolera One using SNAP Biotage columns. NMR spectra were recorded on the BRUKER Ascend 400 at the Department of Chemistry NMR Facility, University of Chicago, for ^{1}H-400 MHz and ^{13}C-101 MHz measurements. Chemical shifts are given in parts per million (δ) referenced to TMS (δ = 0.00 ppm 1H-, 13C-NMR). Coupling constants are given in Hertz. High resolution mass spectra measurements were performed on an Agilent 6224 TOF. Using a combination of atmospheric pressure chemical ionization and electrospray ionization at the Department of Chemistry Mass Spectrometry Facility, University of Chicago.

Boc-Rhodol [6] was synthesized according to the literature.

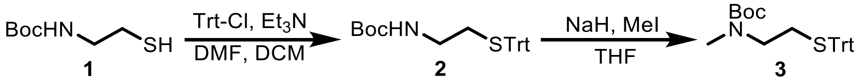

Synthesis of 3. Compound **1** (1.05 g, 1.0 eq, 1.38 mmol) was dissolved 25 mL DCM/DMF (1:1) solution. Trityl chloride (1.65 g, 1.1 eq, 1.38 mmol) and triethylamine (1.05 g, 1.0 eq, 1.38 mmol) was added, and the solution was stirred at room temperature for 2 hours. Then the mixture was concentrated in vacuo and the crude product was dissolved in 100 mL DCM and washed with saturated $NaHCO_3$ solution and Brine. The organic phase was dried, filtered and concentrated in vacuo. The product **2** was used for the next step without further purification. NaH (1.05 g, 1.0 eq, 1.38 mmol) was suspended in 25 mL dry THF at 0 ℃ under nitrogen. Compound **2** in 10 mL dry was added dropwise. The reaction mixture was stirred for 15 min, followed by the addition of MeI (1.05 g, 1.0 eq, 1.38 mmol). The ice bath was removed after 30 min and the reaction mixture was stirred at room temperature overnight. THF was removed by rotary evaporation, and the crude

product was diluted with ethyl acetate and washed with water and brine. The organic phase was dried with Na_2SO_4, filtered, and concentrated. The resultant crude product was purified by column chromatography (Silica; 0-100% DCM:hexane) to yield compound **3** (1.77 g, 67% over two steps) as a colorless oil. R_f: 0.61 (Silica; DCM).

^{1}H NMR (400 MHz, $CDCl_3$): δ 7.49 – 7.39 (m, 6H), 7.30 (dd, J = 8.6, 6.8 Hz, 6H), 7.25 – 7.19 (m, 3H), 3.06 (t, J = 7.5 Hz, 2H), 2.71 – 2.53 (m, 3H), 2.37 (d, J = 8.3 Hz, 2H), 1.43 (s, 9H).

^{13}C NMR (101 MHz, $CDCl_3$): δ 155.39, 144.83, 129.65, 127.94, 126.72, 79.51, 66.74, 48.06, 34.71, 34.53, 30.23, 28.46.

HRA-MS (+): Calculated for $C_{27}H_{31}NO_2S$ [M+] 433.2075; found 433.2094.

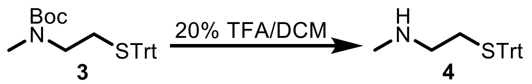

Synthesis of 4. A solution of compound **3** (1.77 g, 1.0 eq, 4.09 mmol) in 20% TFA in DCM (20 mL) was stirred for 1 hour at room temperature. Once Boc deprotection was complete, the reaction was diluted with DCM (3x 20 mL) and concentrated via rotary evaporation. The resultant crude product was purified by column chromatography (Silica; 0-10% MeOH:DCM) to yield compound **4** (1.30 g, 95%) as a white solid. R_f: 0.45 (Silica; 10% MeOH in DCM).

^{1}H NMR (400 MHz, $CDCl_3$): δ 9.16 (s, 2H), 7.47 – 7.35 (m, 6H), 7.31 – 7.23 (m, 6H), 7.23 – 7.17 (m, 3H), 2.58 (dd, J = 8.9, 6.3 Hz, 2H), 2.32 (t, J = 7.7 Hz, 2H), 2.24 (s, 3H).

^{13}C NMR (101 MHz, $CDCl_3$): δ 144.09, 129.50, 128.20, 127.07, 67.49, 47.76, 32.42, 27.08.

HRA-MS (+): Calculated for $C_{22}H_{23}NS$ [M+] 333.1551; found 333.1559.

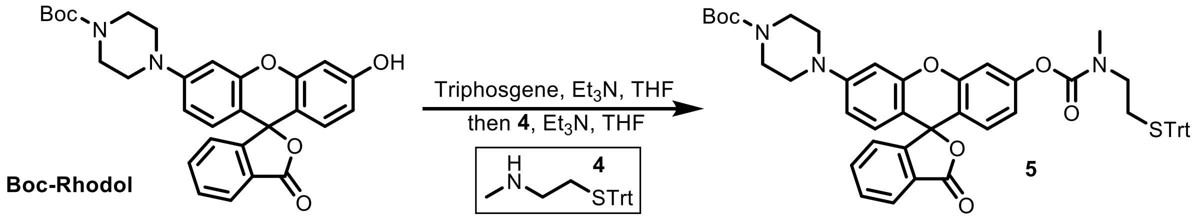

Synthesis of 5. To a solution of triphosgene (440 mg, 1 eq, 1.48 mmol) in dry THF (20 mL) at 0°C, a solution of Boc-Rhodol (740 mg, 1 eq, 1.48 mmol) with triethylamine (1.85 mL; 0.80 M in THF; 1 eq) in dry THF (10 mL) was added dropwise over 20 min. The ice bath was then replaced by a 40 °C water bath, and nitrogen was flushed through to evaporate the THF. Dry THF (20 mL)

was freshly added to the reaction mixture, followed by slow addition of compound **4** (460.0 mg, 0.93 eq, 1.38 mmol) in 8 mL of THF. This was followed by the dropwise addition of triethylamine (1.76 mL; 0.80 M in THF, 0.95 eq) until the reaction was complete by LC-MS. The reaction mixture was evaporated, and the crude product was purified by column chromatography (Silica; 0-75% EtOAc:hexane) to yield compound **5** (802 mg, 65%) as light red oil. R_f: 0.61 (Silica; 50% EtOAc:hexane).

^{1}H NMR (400 MHz, $CDCl_3$): δ 8.02 (dd, J = 7.3, 3.9 Hz, 1H), 7.70 – 7.56 (m, 2H), 7.47 – 7.38 (m, 6H), 7.34 – 7.24 (m, 6H), 7.21 (tdd, J = 7.4, 4.8, 1.5 Hz, 3H), 7.19 – 7.12 (m, 1H), 7.07 – 6.96 (m, 1H), 6.80 – 6.56 (m, 5H), 3.65 – 3.52 (m, 4H), 3.22 (q, J = 4.6 Hz, 5H), 3.12 (t, J = 7.4 Hz, 1H), 2.80 (d, J = 14.1 Hz, 3H), 2.51 (q, J = 7.0 Hz, 2H), 1.49 (s, 9H).

^{13}C NMR (101 MHz, $CDCl_3$): δ 169.48, 154.68, 153.69, 153.15, 152.81, 152.66, 152.61, 152.31, 151.95, 144.71, 144.62, 135.00, 129.73, 129.67, 129.60, 128.78, 128.38, 128.02, 127.99, 126.86, 126.79, 126.66, 125.01, 124.09, 117.47, 117.38, 116.19, 112.32, 110.34, 110.23, 109.38, 102.40, 82.88, 80.11, 67.07, 67.02, 48.90, 48.54, 48.23, 35.27, 35.20, 30.20, 29.63, 28.45.

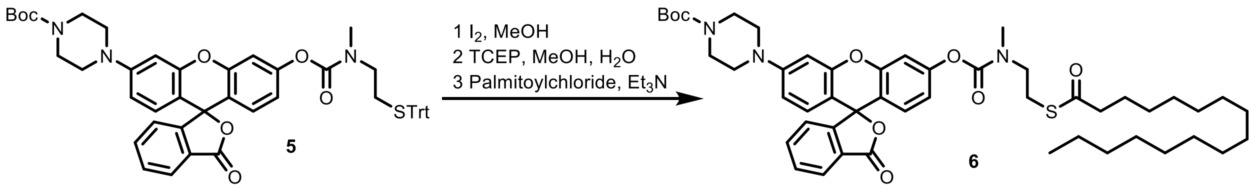

Synthesis of 6. Compound **5** (300.0 mg, 1.0 eq, 0.35 mmol) was dissolved in 15 mL MeOH, then I_2-MeOH solution was slowly added (for 1 eq I_2, add 88.8 mg I_2 to 1 mL MeOH) until the reaction was complete by LC-MS. The reaction mixture was then quenched with a solution of 0.2 M sodium citrate and 0.2 M sodium ascorbate at pH ~3-4 until the yellow color disappeared. The quenched reaction mixture was diluted with DCM (50 mL) and washed with brine (30 mL). The aqueous layer was washed again with DCM (25 mL). The combined organic layers were dried over Na_2SO_4 and solvent was removed by rotary evaporation. The resultant crude product containing the disulfide was resuspended in 9:1 MeOH: H2O (10 mL), and TCEP•HCl (300 mg, 3 eq, 1.05 eq) was added. The reaction mixture was stirred at room temperature for 30 min until the reaction was complete by LC-MS. The reaction mixture was diluted with 50 mL DCM and washed with brine (30 mL). The aqueous layer was washed again with DCM (25 mL). The combined organic layers were dried over Na_2SO_4 and solvent was removed by rotary evaporation.

The resultant crude material was dissolved in 10 mL DCM, then palmitoyl chloride (320 µL, 3 eq, 1.05 mmol) was added dropwise, followed by slow addition of triethylamine until the reaction was complete by LC-MS. The reaction mixture was then diluted with 20 mL DCM and quenched with 30 mL 5% HCl. The aqueous layer was washed again with DCM (20 mL). The combined organic layers were dried over Na_2SO_4 and solvent was removed by rotary evaporation. The resultant crude product was purified by column chromatography (Silica; 0-50% EtOAc:hexane) to yield compound **6** (167 mg, 56%) as near white solid. R_f: 0.78 (Silica; 50% EtOAc:hHexane).

^{1}H NMR (400 MHz, $CDCl_3$): δ 8.05 – 7.99 (m, 1H), 7.69 – 7.56 (m, 2H), 7.15 (d, J = 7.4 Hz, 1H), 7.10 (dd, J = 5.8, 2.2 Hz, 1H), 6.86 – 6.73 (m, 2H), 6.71 – 6.63 (m, 2H), 6.60 (dd, J = 8.9, 2.4 Hz, 1H), 3.57 (t, J = 5.2 Hz, 5H), 3.49 (t, J = 7.1 Hz, 1H), 3.21 (t, J = 5.3 Hz, 4H), 3.16 – 3.10 (m, 4H), 3.05 (s, 1H), 2.56 (td, J = 7.6, 5.8 Hz, 2H), 1.64 (p, J = 7.2 Hz, 2H), 1.48 (s, 9H), 1.37 – 1.13 (m, 24H), 0.87 (t, J = 6.8 Hz, 3H).

^{13}C NMR (101 MHz, $CDCl_3$): δ 199.28, 198.93, 169.44, 154.66, 154.03, 153.76, 153.15, 152.82, 152.64, 152.29, 151.99, 134.97, 129.71, 128.88, 128.77, 128.36, 126.66, 125.01, 124.07, 117.38, 116.25, 112.30, 110.26, 110.21, 109.34, 102.38, 82.83, 80.08, 77.24, 49.16, 48.77, 48.21, 44.24, 35.51, 35.47, 31.93, 29.70, 29.67, 29.64, 29.59, 29.41, 29.36, 29.24, 28.95, 28.43, 26.92, 26.33, 25.62, 25.60, 22.70, 14.13.

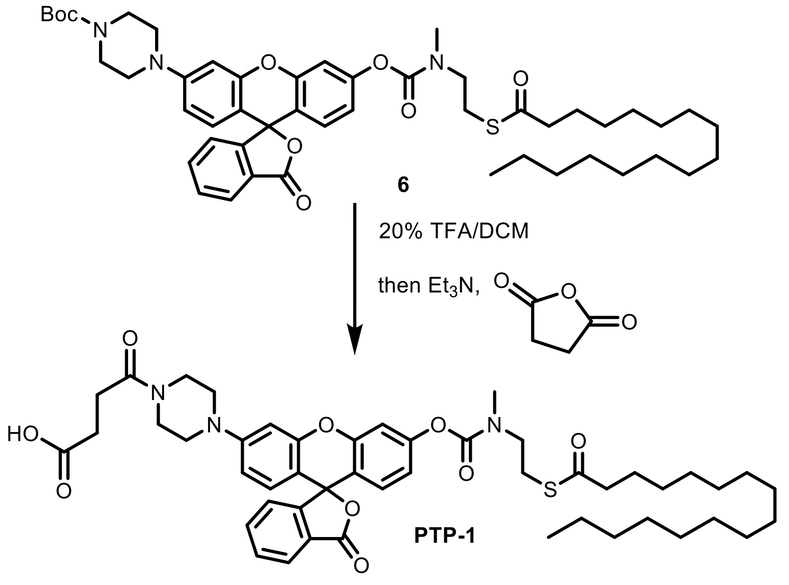

Synthesis of PTP-1. A solution of compound **6** (50 mg, 1.0 eq, 58 µmol) in 20% TFA in DCM (5 mL) was stirred for 30 min at room temperature. Once Boc deprotection was complete, the

reaction was diluted with DCM (3x 5 mL) and solvent was removed by rotary evaporation. The resulting crude was suspended in DCM (5 mL) and succinic anhydride (36 mg, 420 µmol, 7.2 eq) was added, followed by slow addition of triethylamine until the reaction was completed. The reaction mix was acidified with 10 mL 1M HCl and diluted with DCM (20 mL) then washed with brine (20 mL). The aqueous layer was washed again with DCM (20 mL) and the combined organic layers were dried over Na_2SO_4 and evaporated by rotary evaporation. Purification by column chromatography (Silica; 0-10% MeOH in DCM) afforded **PTP-1** as a pink solid (40.7 mg, 81%). Rf: 0.32 (Silica; 5% MeOH:DCM)

^{1}H NMR (400 MHz, $CDCl_3$): δ 8.01 (d, J = 7.4 Hz, 1H), 7.69 – 7.56 (m, 2H), 7.15 (d, J = 7.4 Hz, 1H), 7.10 (dd, J = 6.5, 2.3 Hz, 1H), 6.82 (td, J = 8.5, 2.3 Hz, 1H), 6.76 (d, J = 8.6 Hz, 1H), 6.71 – 6.63 (m, 2H), 6.59 (dd, J = 8.9, 2.4 Hz, 1H), 3.77 (t, J = 5.2 Hz, 2H), 3.69 (s, 1H), 3.64 (t, J = 5.2 Hz, 2H), 3.57 (t, J = 7.1 Hz, 1H), 3.48 (t, J = 7.1 Hz, 1H), 3.25 (dt, J = 15.4, 5.3 Hz, 4H), 3.17 – 3.02 (m, 5H), 2.70 (tt, J = 10.5, 5.1 Hz, 4H), 2.56 (td, J = 7.5, 5.6 Hz, 2H), 1.65 (h, J = 7.0 Hz, 2H), 1.24 (s, 24H), 0.87 (t, J = 6.7 Hz, 3H).

^{13}C NMR (101 MHz, $CDCl_3$): δ 199.34, 198.99, 176.87, 176.77, 172.68, 170.30, 169.46, 154.04, 153.78, 153.08, 152.66, 152.38, 152.28, 151.93, 135.04, 129.78, 128.89, 128.86, 127.79, 126.61, 125.05, 124.05, 117.47, 117.43, 116.21, 116.18, 113.95, 112.31, 110.29, 110.24, 109.71, 102.50, 82.77, 77.37, 77.05, 76.73, 51.94, 49.18, 48.80, 48.27, 48.09, 45.00, 44.24, 44.21, 41.52, 35.52, 35.51, 31.93, 29.70, 29.69, 29.67, 29.66, 29.63, 29.58, 29.41, 29.36, 29.24, 28.95, 28.83, 28.71, 27.89, 27.08, 26.91, 26.31, 25.62, 25.60, 22.70, 14.13.

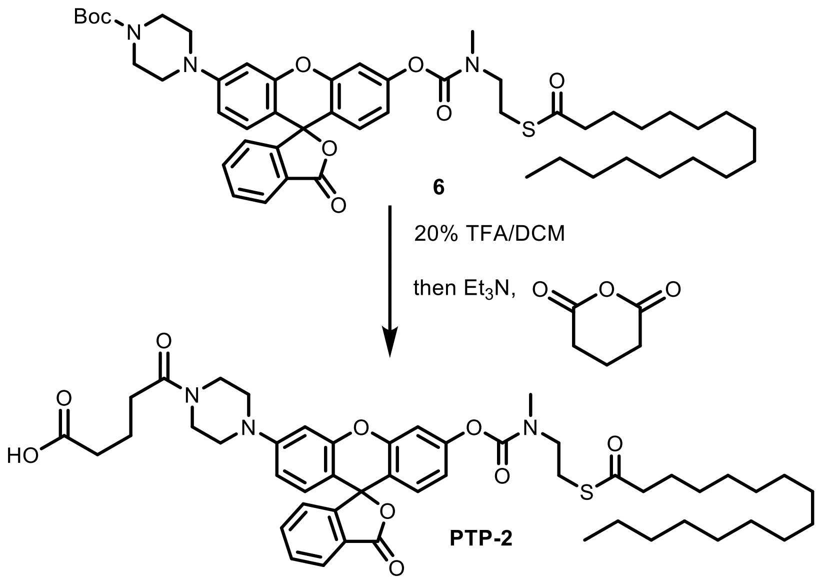

Synthesis of PTP-2. A solution of compound **6** (45.5 mg, 1.0 eq, 53 µmol) in 20% TFA in DCM (5 mL) was stirred for 30 min at room temperature. Once Boc deprotection was complete, the reaction was diluted with DCM (3x 5 mL) and solvent was removed by rotary evaporation. The resulting crude was suspended in DCM (5 mL) and glutaric anhydride (46 mg, 403 µmol, 7.6 eq) was added followed, by slow addition of triethylamine until the reaction was completed. The reaction mix was acidified with 10 mL 1M HCl and diluted with DCM (20 mL) then washed with brine (20 mL). The aqueous layer was washed again with DCM (20 mL) and the combined organic layers were dried over Na_2SO_4 and evaporated by rotary evaporation. Purification by column chromatography (Silica; 0-10% MeOH in DCM) afforded **PTP-2** as a light red oil (24.5 mg, 53%). Rf: 0.35 (Silica; 5% MeOH:DCM)

^{1}H NMR (400 MHz, $CDCl_3$): δ 8.01 (dd, J = 7.0, 1.4 Hz, 1H), 7.68 – 7.57 (m, 2H), 7.14 (d, J = 7.4 Hz, 1H), 7.09 (dd, J = 6.9, 2.3 Hz, 1H), 6.85 – 6.74 (m, 2H), 6.70 – 6.63 (m, 2H), 6.59 (dd, J = 8.9, 2.4 Hz, 1H), 3.75 (q, J = 4.4 Hz, 2H), 3.62 (dd, J = 6.8, 3.8 Hz, 2H), 3.56 (t, J = 7.1 Hz, 1H), 3.48 (dd, J = 8.0, 6.2 Hz, 1H), 3.23 (td, J = 8.1, 3.5 Hz, 4H), 3.17 – 3.00 (m, 5H), 2.55 (td, J = 7.5, 5.7 Hz, 2H), 2.51 – 2.30 (m, 4H), 2.03 – 1.89 (m, 2H), 1.67 – 1.60 (m, 2H), 1.28 – 1.16 (m, 24H), 0.87 (t, J = 6.8 Hz, 3H)..

^{13}C NMR (101 MHz, $CDCl_3$): δ 199.34, 199.00, 177.66, 171.11, 169.49, 154.05, 153.79, 153.07, 152.65, 152.44, 152.27, 151.94, 135.06, 129.79, 128.89, 128.84, 127.79, 126.61, 125.04, 124.05, 117.42, 116.21, 116.18, 113.94, 112.29, 110.29, 110.25, 109.62, 102.46, 82.81, 77.39, 77.07, 76.76, 60.48, 49.18, 48.79, 48.42, 48.13, 45.11, 44.24, 44.21, 41.27, 35.52, 33.12, 32.05, 31.93, 29.70, 29.67, 29.65, 29.64, 29.58, 29.41, 29.36, 29.24, 28.94, 26.90, 26.30, 25.62, 25.59, 24.73, 22.70, 20.16, 14.14..

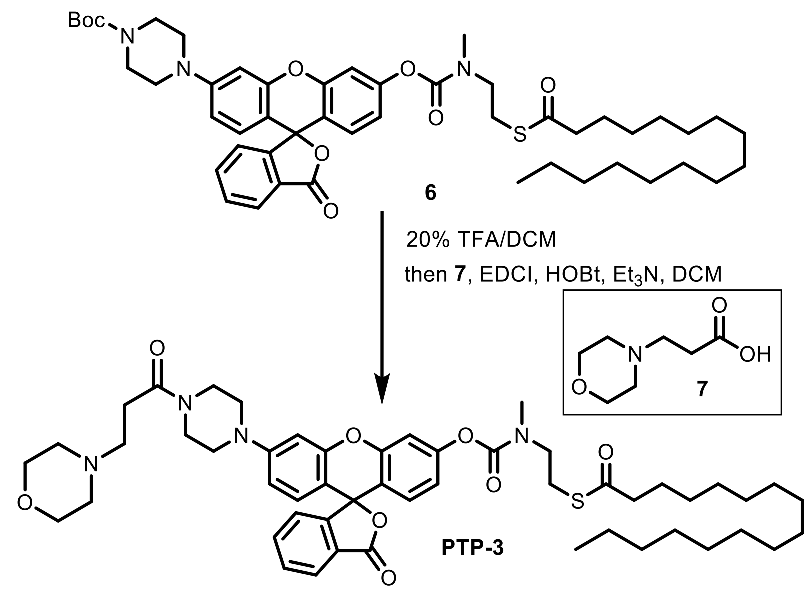

Synthesis of PTP-3. A solution of compound **6** (57.7 mg, 1.0 eq, 67 µmol) in 20% TFA in DCM (5 mL) was stirred for 30 min at room temperature. Once Boc deprotection was complete, the reaction was diluted with DCM (3x 5 mL) and solvent was removed by rotary evaporation. A solution of **7** (51 mg, 4.8 eq, 0.32 mmol), EDCI (61 mg, 4.8 eq, 0.32 mmol), HOBt (43 mg, 4.8 eq, 0.32 mmol) and triethylamine (18.0 µL, 2.0 eq, 0.13 mmol) in 10 mL dry DCM was stirred at room temperature for 10 min. Then the deprotected **6** in 5 mL of DCM was transferred to the reaction and stirred at room temperature until the reaction was complete by LC-MS. The reaction was then diluted with 30 mL of DCM and washed with Sat. $NaHCO_3$. The aqueous layer was washed again with 20 mL DCM, and the combined organic layers were dried with Na_2SO_4, filtered, and concentrated. The resultant crude product was purified by column chromatography (Silica; 0-8% MeOH:DCM) to yield **PTP-3** (35.7 mg, 59%) as colorless oil. R_f: 0.44 (Silica; 5% MeOH:DCM).

^{1}H NMR (400 MHz, $CDCl_3$): δ 8.05 – 7.98 (m, 1H), 7.67 – 7.58 (m, 2H), 7.15 (d, J = 7.4 Hz, 1H), 7.12 – 7.07 (m, 1H), 6.85 – 6.79 (m, 1H), 6.76 (dd, J = 8.7, 1.3 Hz, 1H), 6.71 – 6.64 (m, 2H), 6.60 (dd, J = 8.9, 2.4 Hz, 1H), 3.76 (d, J = 6.0 Hz, 2H), 3.74 – 3.68 (m, 4H), 3.63 (d, J = 5.5 Hz, 2H), 3.57 (t, J = 7.1 Hz, 1H), 3.49 (t, J = 7.1 Hz, 1H), 3.24 (dt, J = 11.8, 5.3 Hz, 4H), 3.17 – 3.08 (m, 3H), 3.05 (s, 1H), 2.75 (dd, J = 8.5, 6.5 Hz, 2H), 2.57 (ddd, J = 10.7, 7.8, 5.7 Hz, 4H), 2.50 (t, J = 4.7 Hz, 4H), 1.70 – 1.59 (m, 3H), 1.24 (t, J = 2.5 Hz, 24H), 0.87 (t, J = 6.8 Hz, 3H).

^{13}C NMR (101 MHz, $CDCl_3$): δ 199.28, 198.93, 170.10, 169.41, 156.28, 154.01, 153.74, 153.09, 152.67, 152.45, 152.30, 151.94, 143.66, 135.01, 129.76, 128.89, 128.86, 127.79, 126.63, 125.04, 124.05, 117.46, 117.41, 116.21, 113.94, 112.29, 110.27, 110.22, 109.75, 102.49, 82.72, 77.24, 68.78, 68.68, 66.90, 54.46, 53.77, 49.18, 48.79, 48.53, 48.20, 45.16, 44.24, 44.21, 41.74, 41.18,

35.52, 35.49, 31.93, 31.03, 30.72, 29.70, 29.69, 29.66, 29.63, 29.58, 29.41, 29.36, 29.24, 28.95, 26.91, 26.32, 25.62, 25.60, 22.70, 14.13.

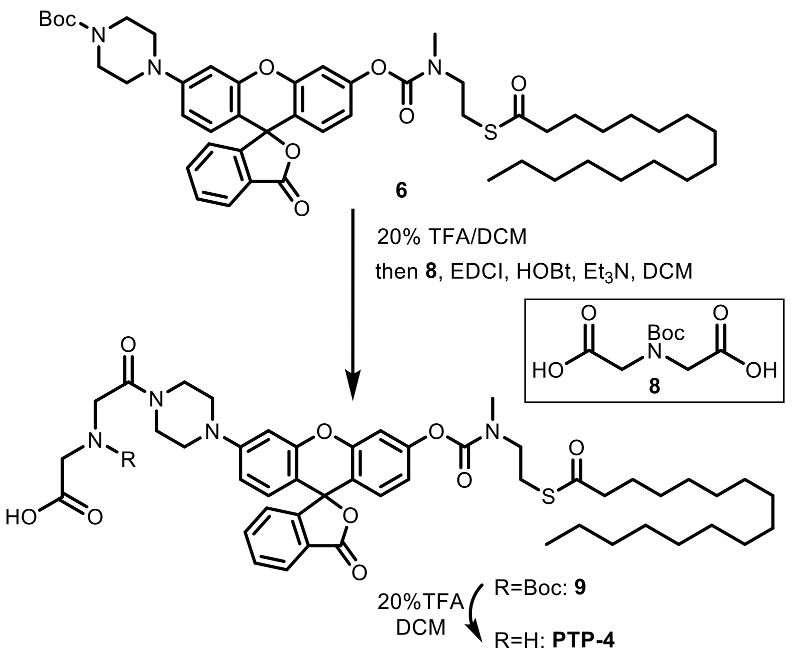

Synthesis of PTP-4. A solution of compound **6** (59.5 mg, 1.0 eq, 69 µmol) in 20% TFA in DCM (5 mL) was stirred for 30 min at room temperature. Once Boc deprotection was complete, the reaction was diluted with DCM (3x 5 mL) and solvent was removed by rotary evaporation. A solution of **8** (97 mg, 6.0 eq, 0.41 mmol), EDCI (61 mg, 4.6 eq, 0.32 mmol), HOBt (43 mg, 4.6 eq, 0.32 mmol) and triethylamine (19.0 µL, 2.0 eq, 0.13 mmol) in 10 mL dry DCM was stirred at room temperature for 10 min. Then the deprotected **6** in 5 mL of DCM was transferred to the reaction and stirred at room temperature until the reaction was complete by LC-MS. The reaction was then diluted with 30 mL of DCM and washed with 5% HCl. The aqueous layer was washed again with 20 mL DCM, and the combined organic layers were dried with Na_2SO_4, filtered, and concentrated. The resultant crude product was purified by column chromatography (Silica; 0-8% MeOH:DCM) to yield **9** (42.3 mg, 63%) as red oil. R_f: 0.41 (Silica; 5% MeOH:DCM).

^{1}H NMR (400 MHz, $CDCl_3$): δ 8.04 – 7.98 (m, 1H), 7.70 – 7.57 (m, 2H), 7.18 – 7.07 (m, 2H), 6.87 – 6.71 (m, 2H), 6.71 – 6.63 (m, 2H), 6.57 (dd, J = 8.9, 2.5 Hz, 1H), 4.25 (s, 2H), 4.19 – 4.03 (m, 1H), 3.91 (s, 2H), 3.80 (dq, J = 6.2, 3.3 Hz, 2H), 3.63 – 3.52 (m, 3H), 3.47 (q, J = 7.7 Hz, 1H), 3.37 – 3.20 (m, 5H), 3.17 – 3.07 (m, 3H), 3.05 (s, 1H), 2.56 (td, J = 7.5, 5.5 Hz, 2H), 1.71 – 1.59 (m, 2H), 1.45 (s, 9H), 1.25 (q, J = 4.1 Hz, 24H), 0.87 (t, J = 6.7 Hz, 3H).

^{13}C NMR (101 MHz, $CDCl_3$): δ 199.27, 198.93, 170.89, 169.91, 169.39, 156.28, 154.77, 154.23, 153.99, 153.72, 152.97, 152.73, 152.30, 152.27, 152.09, 151.87, 135.10, 129.85, 128.91, 127.79, 126.58, 125.07, 124.04, 117.57, 117.53, 116.16, 113.94, 112.38, 110.32, 110.28, 110.01, 102.66, 82.72, 82.69, 81.98, 77.25, 68.66, 55.99, 53.81, 50.84, 49.20, 48.81, 47.96, 47.88, 44.46, 44.24, 44.21, 42.37, 41.73, 35.52, 31.92, 31.03, 29.69, 29.67, 29.65, 29.63, 29.58, 29.41, 29.36, 29.23, 28.94, 28.16, 28.04, 26.90, 26.30, 25.61, 25.59, 22.69, 14.13.

A solution of compound **9** (9.7 mg) in 20% TFA in DCM (5 mL) was stirred for 30 min at room temperature. Once Boc deprotection was completed, the reaction was diluted with DCM (3x 5 mL) and solvent was removed by rotary evaporation. The residue was dissolved in 970 µL DMSO to make 10 mM **PTP-4** stock solution that is ready for the PTP assay. To record the NMR spectrum, 25 mg compound 9 is treated with 20% TFA in DCM (5 mL), and stirred for 30 min at room temperature. Once Boc deprotection was completed, the reaction was diluted with DCM (3*5 mL) and solvent was removed by rotary evaporation. The residue was dissolved in MeOH-d4 and subjected to NMR.

^{1}H NMR (400 MHz, CD3OD_SPE) δ 8.14 (d, J = 7.6 Hz, 1H), 7.79 – 7.66 (m, 2H), 7.50 – 7.40 (m, 1H), 7.24 (d, J = 7.4 Hz, 1H), 7.18 – 6.88 (m, 5H), 4.15 (s, 2H), 3.90 (s, 2H), 3.76 – 3.69 (m, 2H), 3.64 (ddd, J = 6.1, 5.1, 1.0 Hz, 2H), 3.56 (dt, J = 6.4, 4.8 Hz, 5H), 3.51 – 3.41 (m, 2H), 3.21 (p, J = 1.7 Hz, 2H), 3.15 – 3.09 (m, 1H), 3.07 (d, J = 3.3 Hz, 2H), 2.94 (s, 1H), 2.47 (dt, J = 15.0, 7.4 Hz, 2H), 1.58 – 1.45 (m, 2H), 1.15 (q, J = 6.7 Hz, 24H), 0.84 – 0.75 (m, 3H).

^{13}C NMR (101 MHz, CD3OD_SPE) δ 199.21, 199.06, 167.38, 167.24, 166.14, 163.69, 153.44, 133.90, 130.41, 129.47, 110.03, 109.86, 72.18, 71.06, 70.11, 66.75, 66.70, 62.28, 60.79, 48.78, 48.45, 48.26, 48.12, 48.05, 47.84, 47.63, 47.41, 47.20, 46.99, 46.73, 46.53, 46.48, 43.51, 43.48, 43.39, 42.46, 42.37, 41.08, 34.55, 34.46, 31.68, 29.40, 29.38, 29.36, 29.33, 29.27, 29.13, 29.11, 29.08, 28.98, 28.95, 28.56, 26.43, 25.81, 25.39, 25.32, 22.35, 13.08, 12.94.

Compound 3

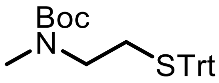
Boc
N
STrt

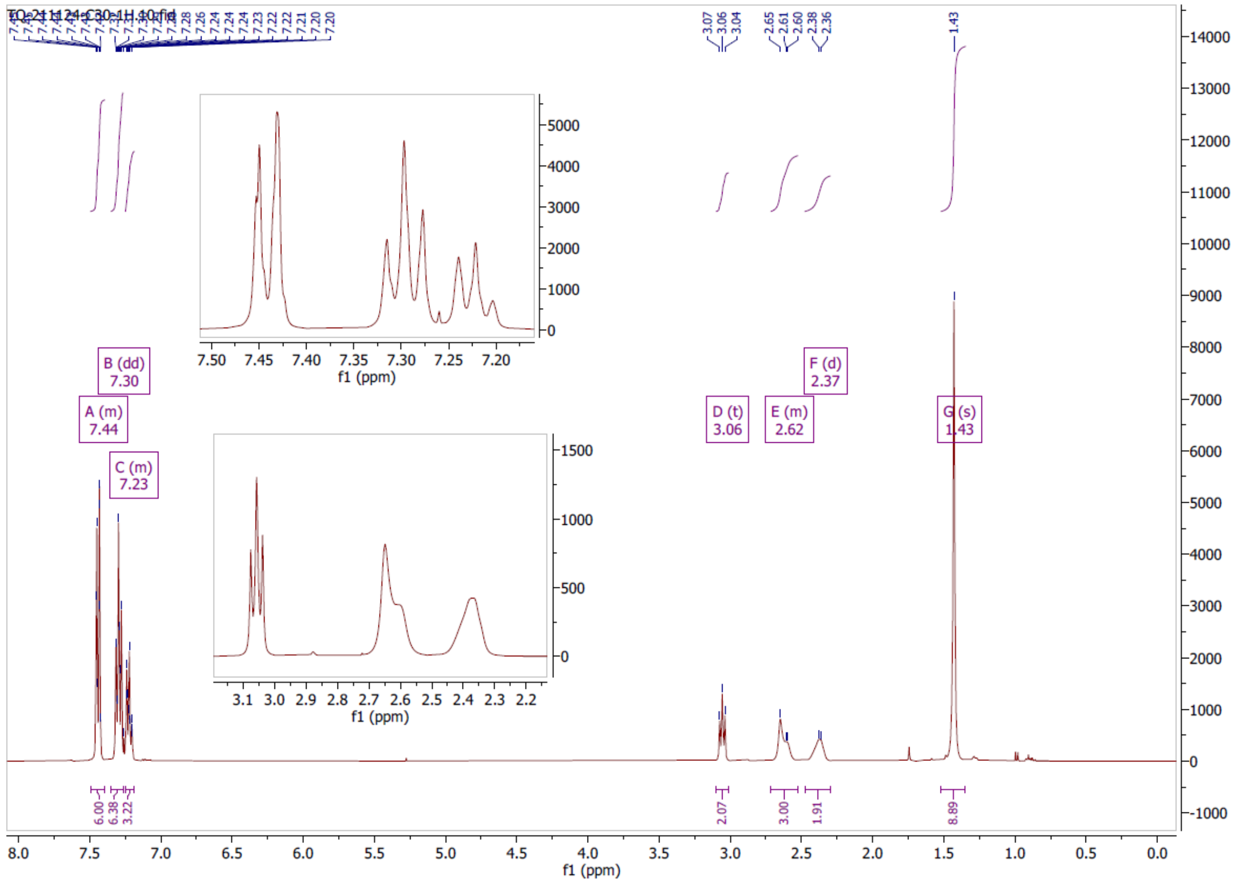
A (m)
7.44
B (dd)
7.30
C (m)
7.23
D (t)
3.06
E (m)
2.62
F (d)
2.37
G (s)
1.43
6.00
6.38
3.22
2.07
3.00
1.91
8.89
f1 (ppm)

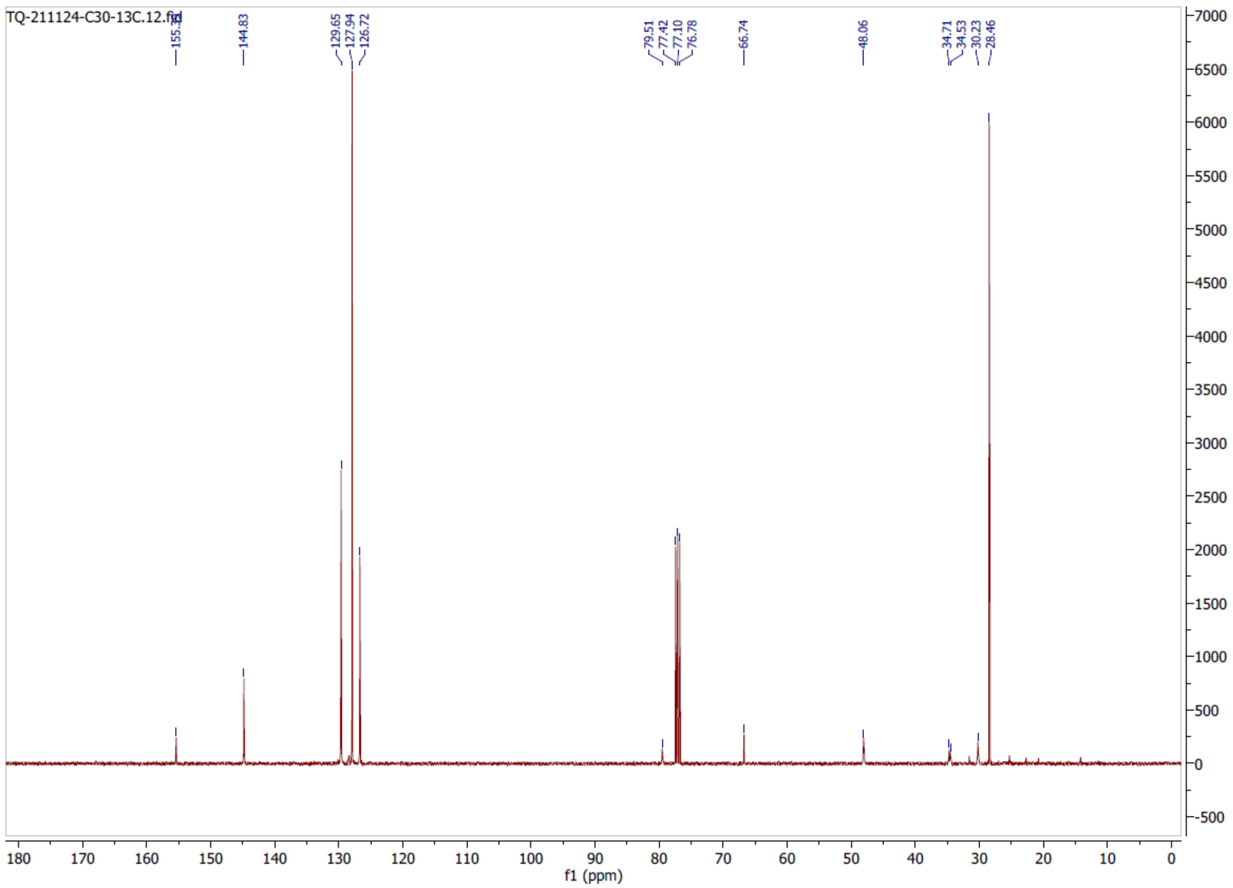
TQ-211124-C30-13C.12.fid
155.[illegible]
144.83
129.65
127.94
126.72
79.51
77.42
77.10
76.78
66.74
48.06
34.71
34.53
30.23
28.46
f1 (ppm)

Compound 4

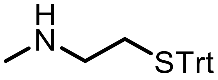
H
N
STrt

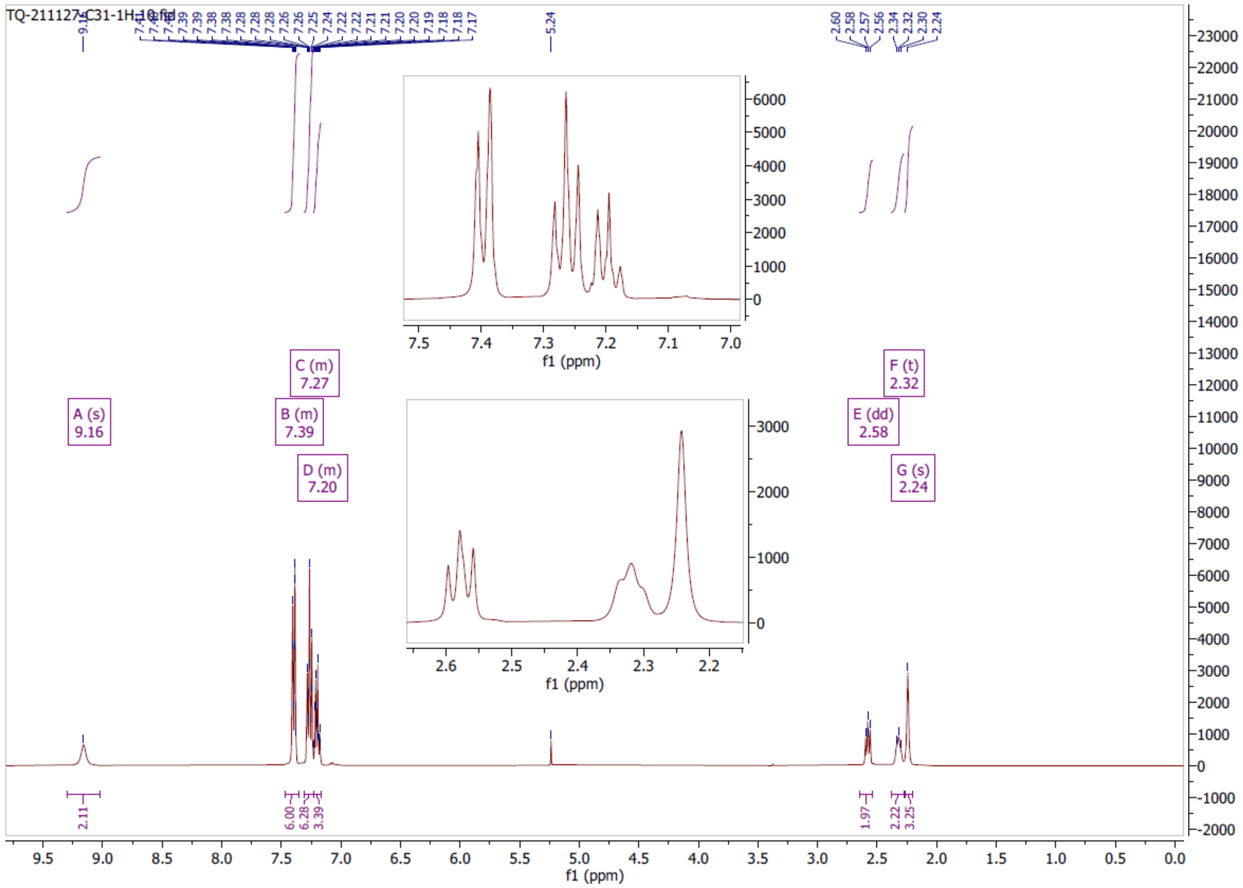
TQ-211127-C31-1H-10.fid
9.16
7.41
7.40
7.40
7.39
7.39
7.38
7.38
7.28
7.28
7.28
7.26
7.26
7.25
7.24
7.22
7.22
7.22
7.21
7.21
7.20
7.20
7.19
7.18
7.18
7.17
5.24
2.60
2.58
2.57
2.56
2.34
2.32
2.30
2.24
A (s)
9.16
B (m)
7.39
C (m)
7.27
D (m)
7.20
E (dd)
2.58
F (t)
2.32
G (s)
2.24
2.11
6.00
6.28
3.39
1.97
2.22
3.25
f1 (ppm)

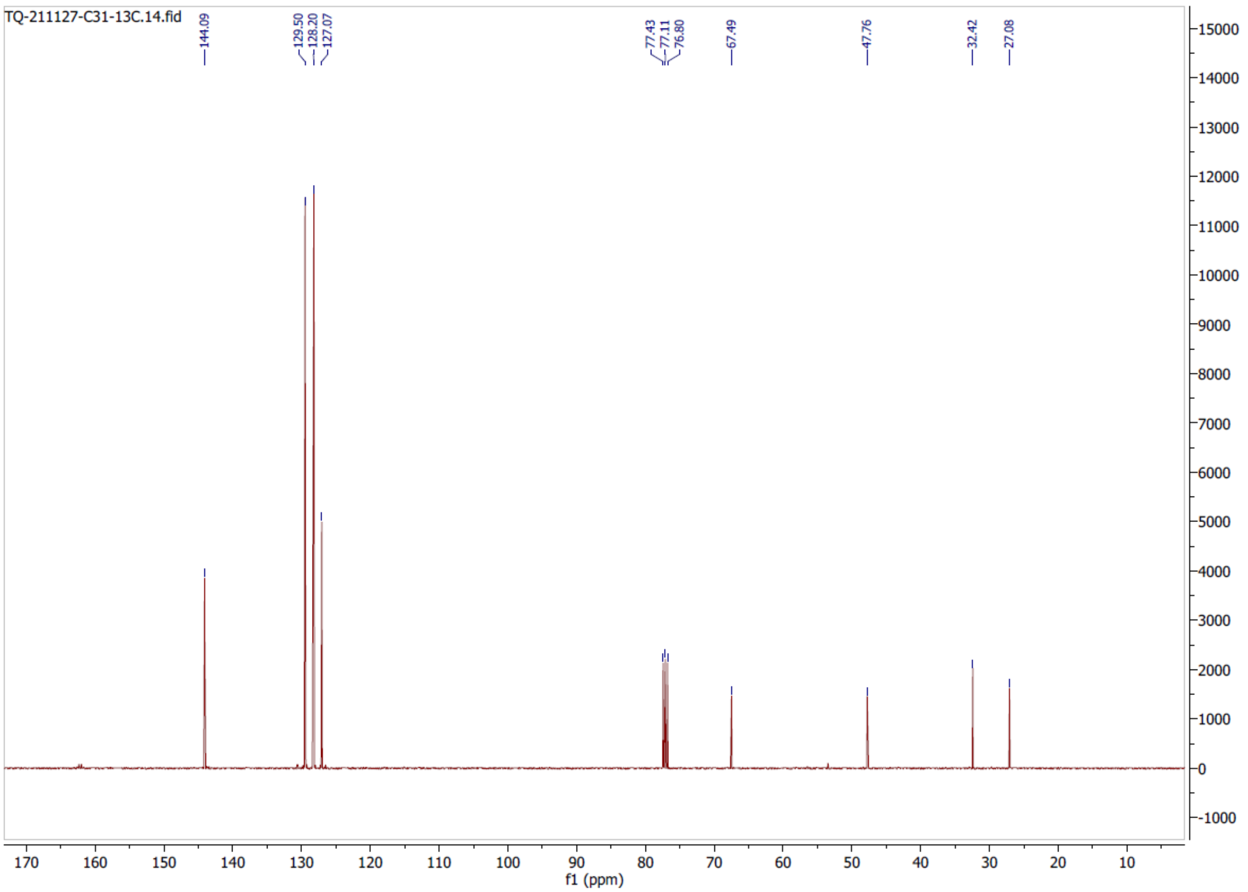
TQ-211127-C31-13C.14.fid
144.09
129.50
128.20
127.07
77.43
77.11
76.80
67.49
47.76
32.42
27.08
15000
14000
13000
12000
11000
10000
9000
8000
7000
6000
5000
4000
3000
2000
1000
0
-1000
170
160
150
140
130
120
110
100
90
80
70
60
50
40
30
20
10
f1 (ppm)

Compound 5

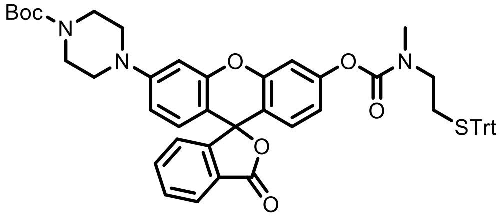
Boc
N
N
O
O
N
O
O
O
STrt

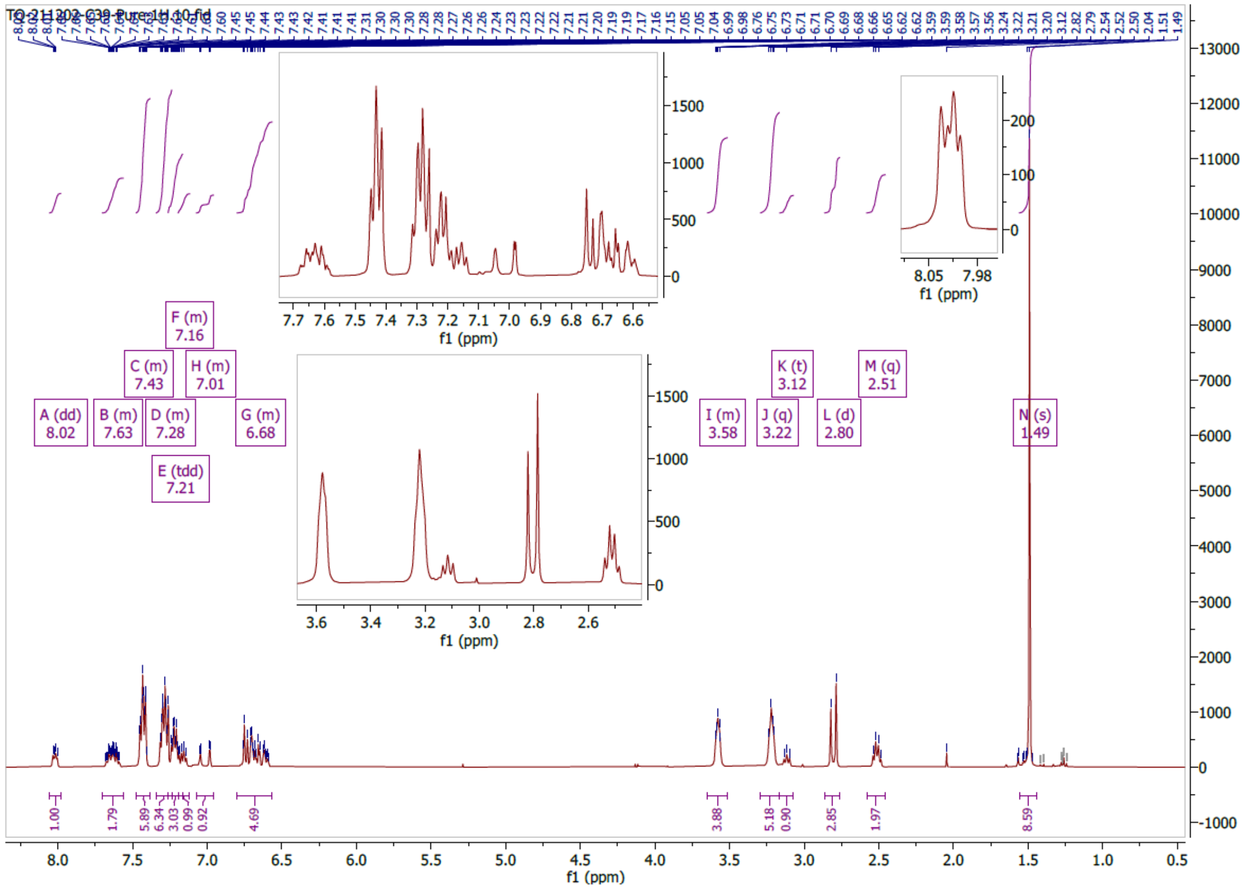
TQ-211202-C39-Pure-1H-10.fid
A (dd)
8.02
B (m)
7.63
C (m)
7.43
D (m)
7.28
E (tdd)
7.21
F (m)
7.16
H (m)
7.01
G (m)
6.68
I (m)
3.58
J (q)
3.22
K (t)
3.12
L (d)
2.80
M (q)
2.51
N (s)
1.49
1.00
1.79
5.89
6.34
3.03
0.99
0.92
4.69
3.88
5.18
0.90
2.85
1.97
8.59
f1 (ppm)

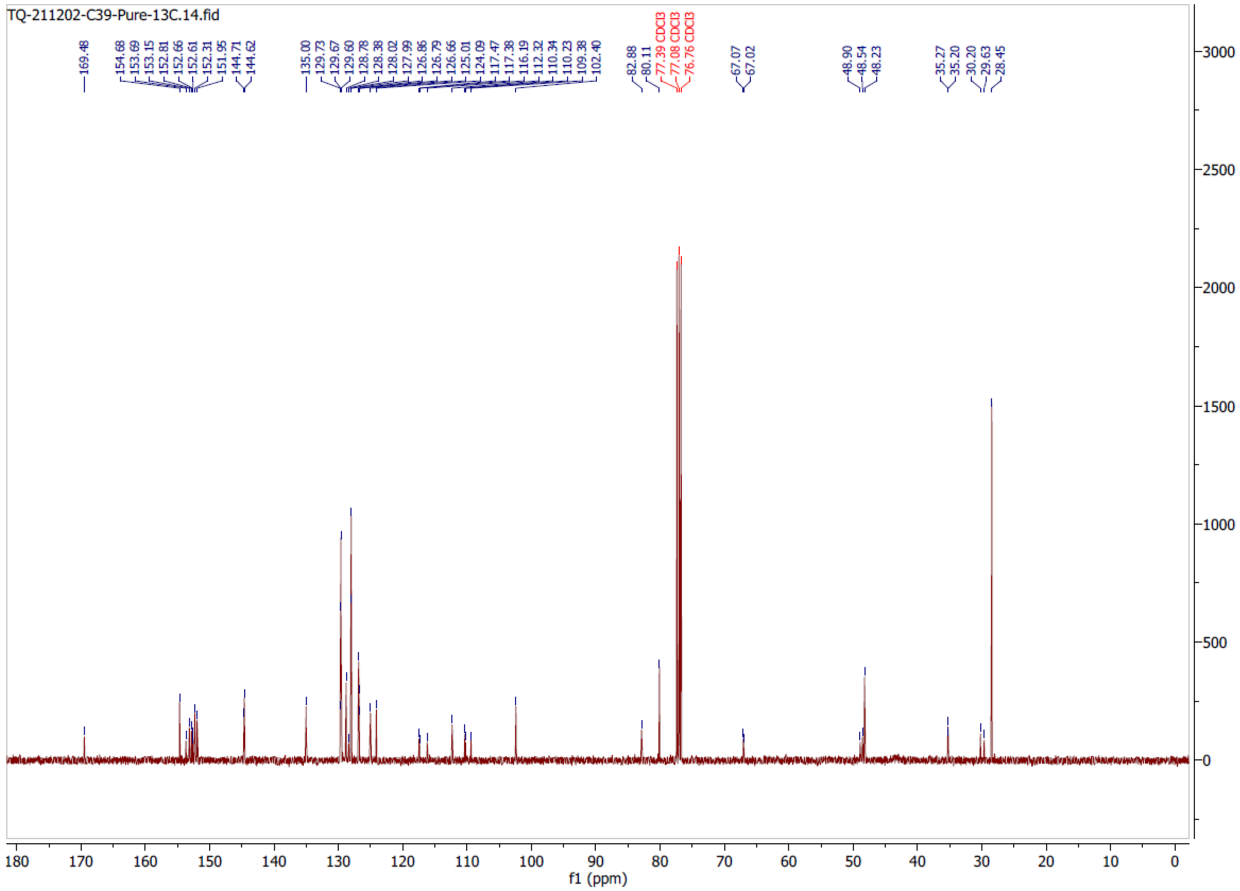
TQ-211202-C39-Pure-13C.14.fid
169.48
154.68
153.69
153.15
152.81
152.66
152.61
152.31
151.95
144.71
144.62
135.00
129.73
129.67
129.60
128.78
128.38
128.02
127.99
126.86
126.79
126.66
125.01
124.09
117.47
117.38
116.19
112.32
110.34
110.23
109.38
102.40
82.88
80.11
77.39 CDCl3
77.08 CDCl3
76.76 CDCl3
67.07
67.02
48.90
48.54
48.23
35.27
35.20
30.20
29.63
28.45
f1 (ppm)

Compound 6

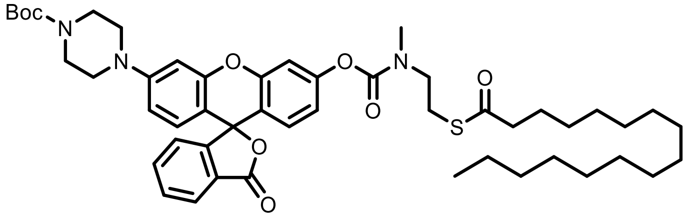

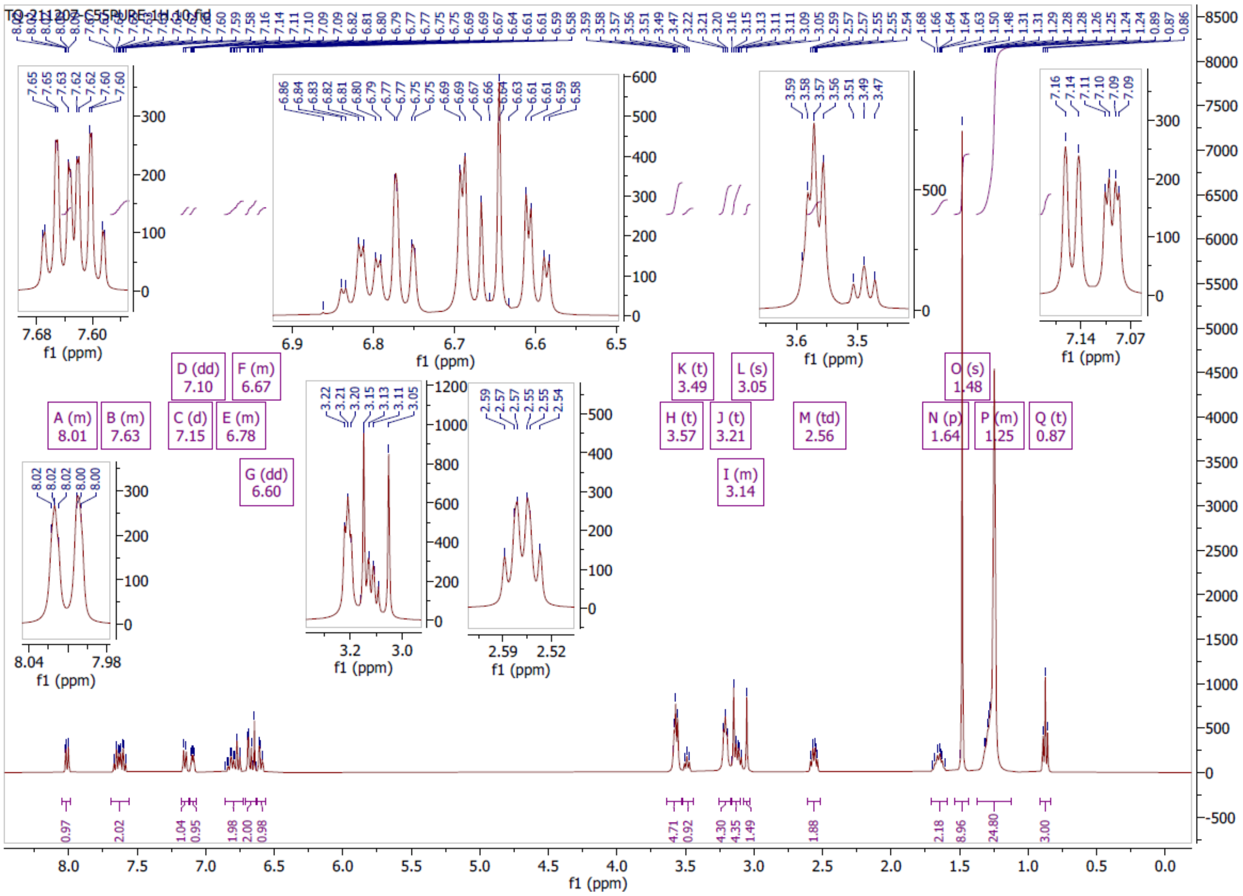

TQ-211207-C55PURE-1H.10.fid
A (m) 8.01
B (m) 7.63
C (d) 7.15
D (dd) 7.10
E (m) 6.78
F (m) 6.67
G (dd) 6.60
H (t) 3.57
I (m) 3.14
J (t) 3.21
K (t) 3.49
L (s) 3.05
M (td) 2.56
N (p) 1.64
O (s) 1.48
P (m) 1.25
Q (t) 0.87
f1 (ppm)

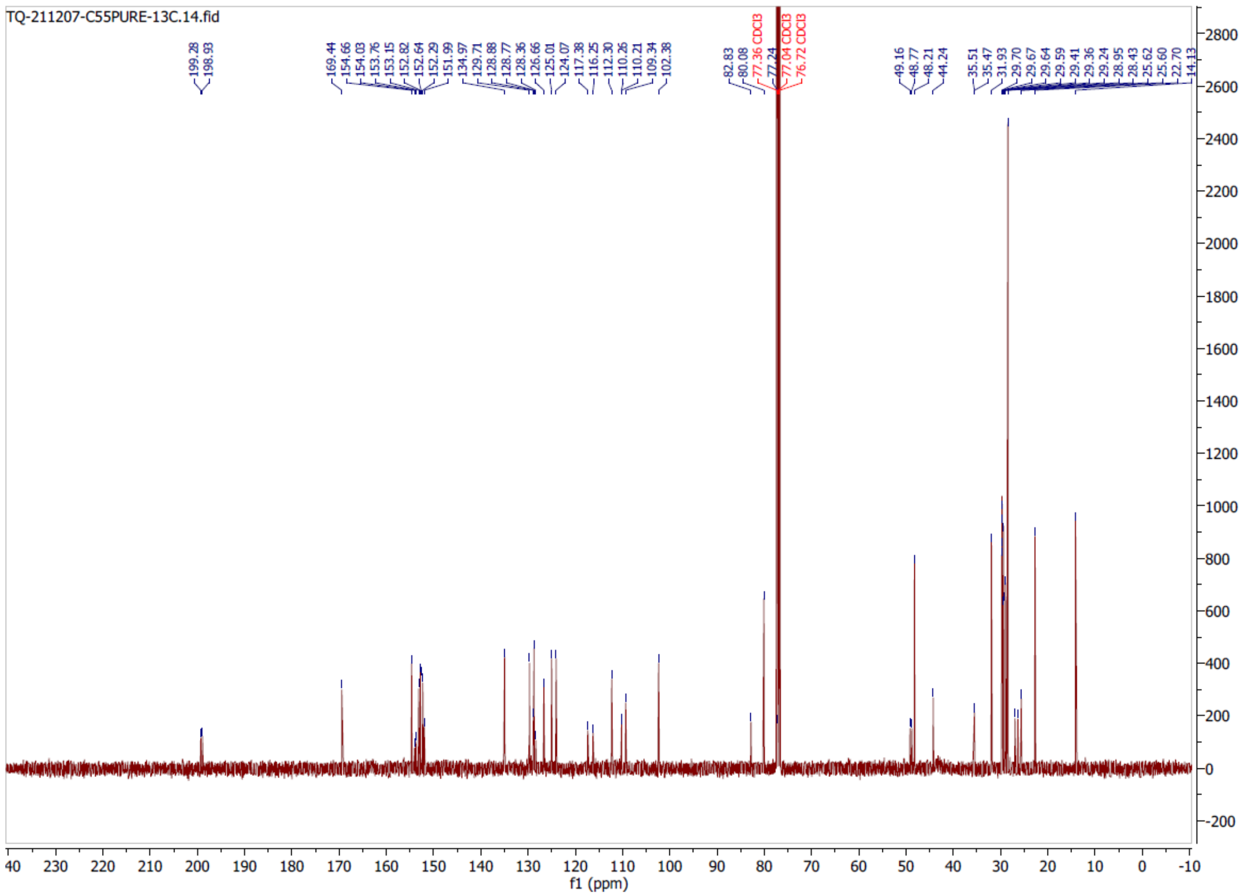
TQ-211207-C55PURE-13C.14.fid
199.28
198.93
169.44
154.66
154.03
153.76
153.15
152.82
152.64
152.29
151.99
134.97
129.71
128.88
128.77
128.36
126.66
125.01
124.07
117.38
116.25
112.30
110.26
110.21
109.34
102.38
82.83
80.08
77.36 CDCl3
77.24
77.04 CDCl3
76.72 CDCl3
49.16
48.77
48.21
44.24
35.51
35.47
31.93
29.70
29.67
29.64
29.59
29.41
29.36
29.24
28.95
28.43
25.62
25.60
22.70
14.13
f1 (ppm)

PTP-1

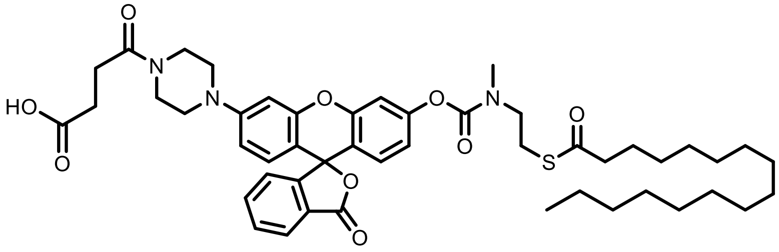

O
N
HO
N
O
O
N
O
O
S
O
O
O

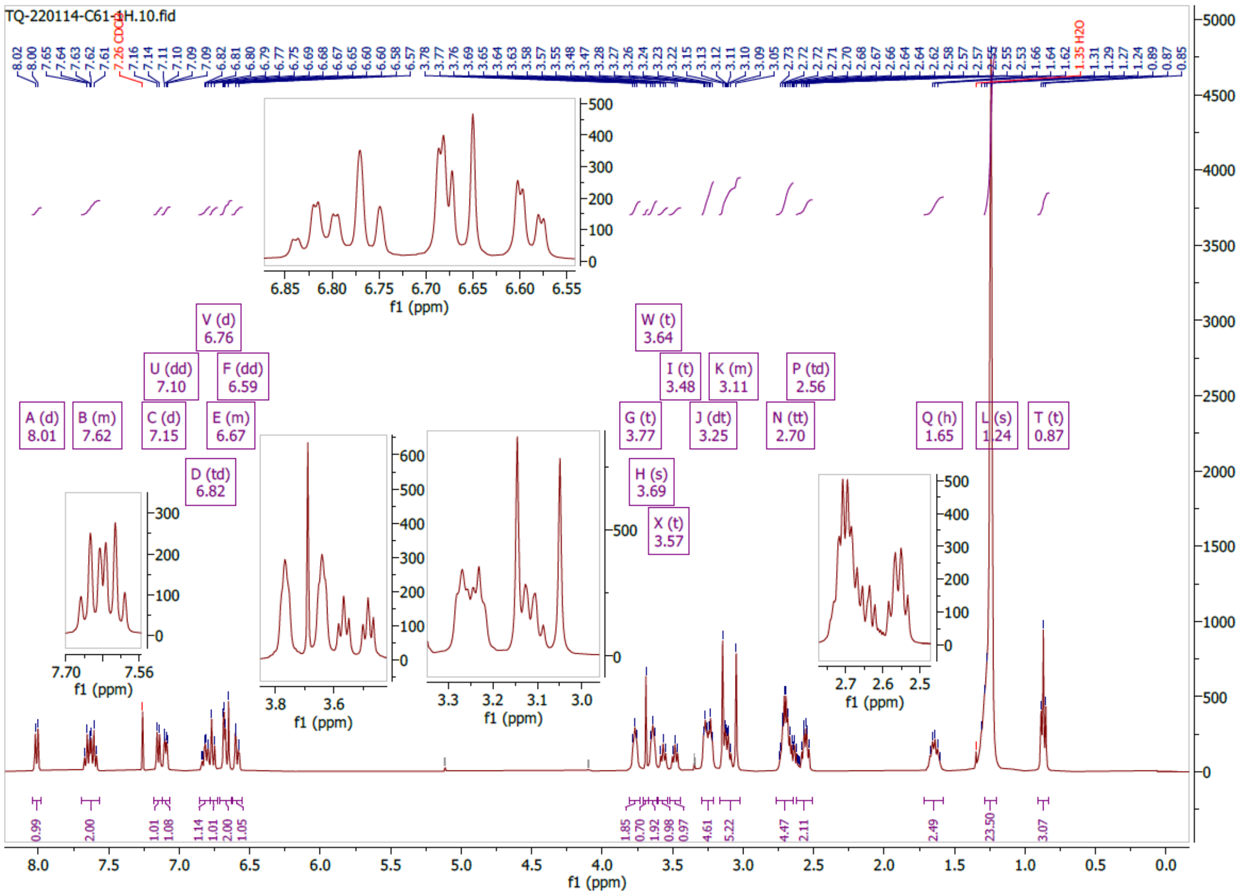

TQ-220114-C61-1H.10.fid
7.26 CDCl3
1.35 H2O
A (d) 8.01
B (m) 7.62
C (d) 7.15
U (dd) 7.10
V (d) 6.76
F (dd) 6.59
E (m) 6.67
D (td) 6.82
G (t) 3.77
W (t) 3.64
H (s) 3.69
X (t) 3.57
I (t) 3.48
J (dt) 3.25
K (m) 3.11
N (tt) 2.70
P (td) 2.56
Q (h) 1.65
L (s) 1.24
T (t) 0.87
f1 (ppm)

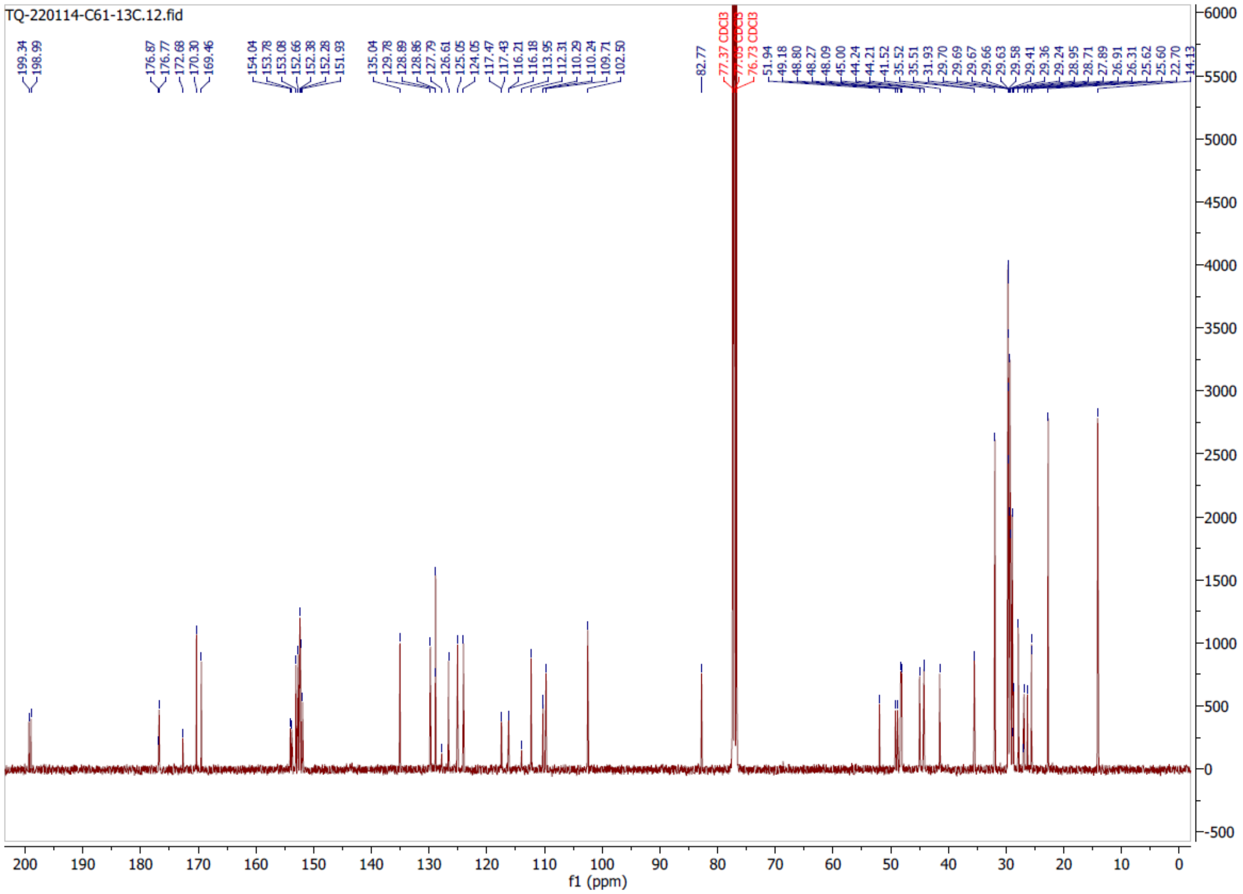
TQ-220114-C61-13C.12.fid
199.34
198.99
176.87
176.77
172.68
170.30
169.46
154.04
153.78
153.08
152.66
152.38
152.28
151.93
135.04
129.78
128.89
128.86
127.79
126.61
125.05
124.05
117.47
117.43
116.21
116.18
113.95
112.31
110.29
110.24
109.71
102.50
82.77
77.37 CDCl3
77.05 CDCl3
76.73 CDCl3
51.94
49.18
48.80
48.27
48.09
45.00
44.24
44.21
41.52
35.52
35.51
31.93
29.70
29.69
29.67
29.66
29.63
29.58
29.41
29.36
29.24
28.95
28.71
27.89
26.91
26.31
25.62
25.60
22.70
14.13
6000
5500
5000
4500
4000
3500
3000
2500
2000
1500
1000
500
0
-500
200
190
180
170
160
150
140
130
120
110
100
90
80
70
60
50
40
30
20
10
0
f1 (ppm)

PTP-2

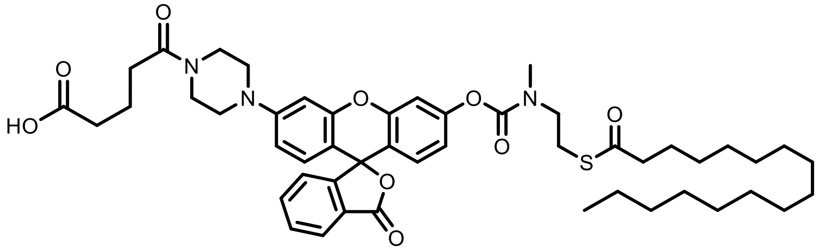

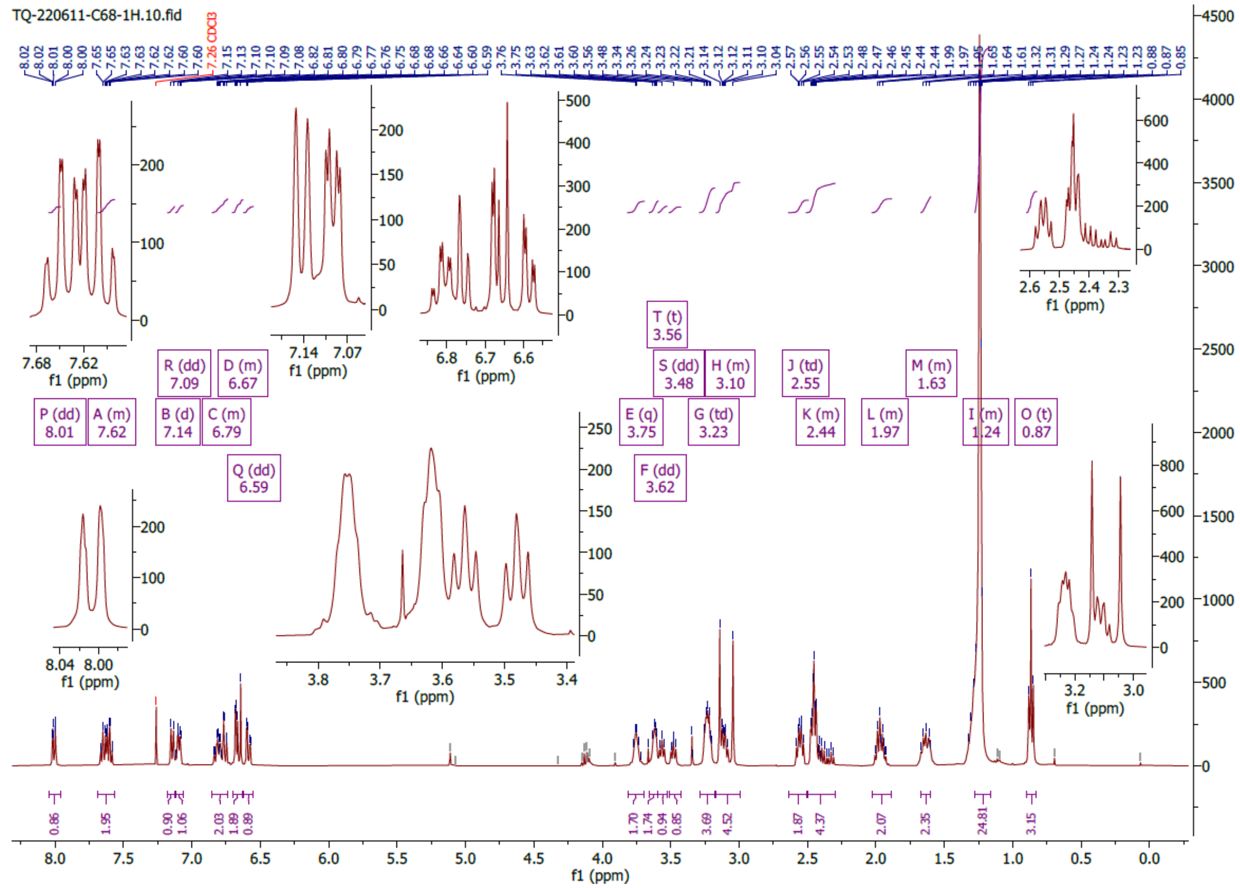

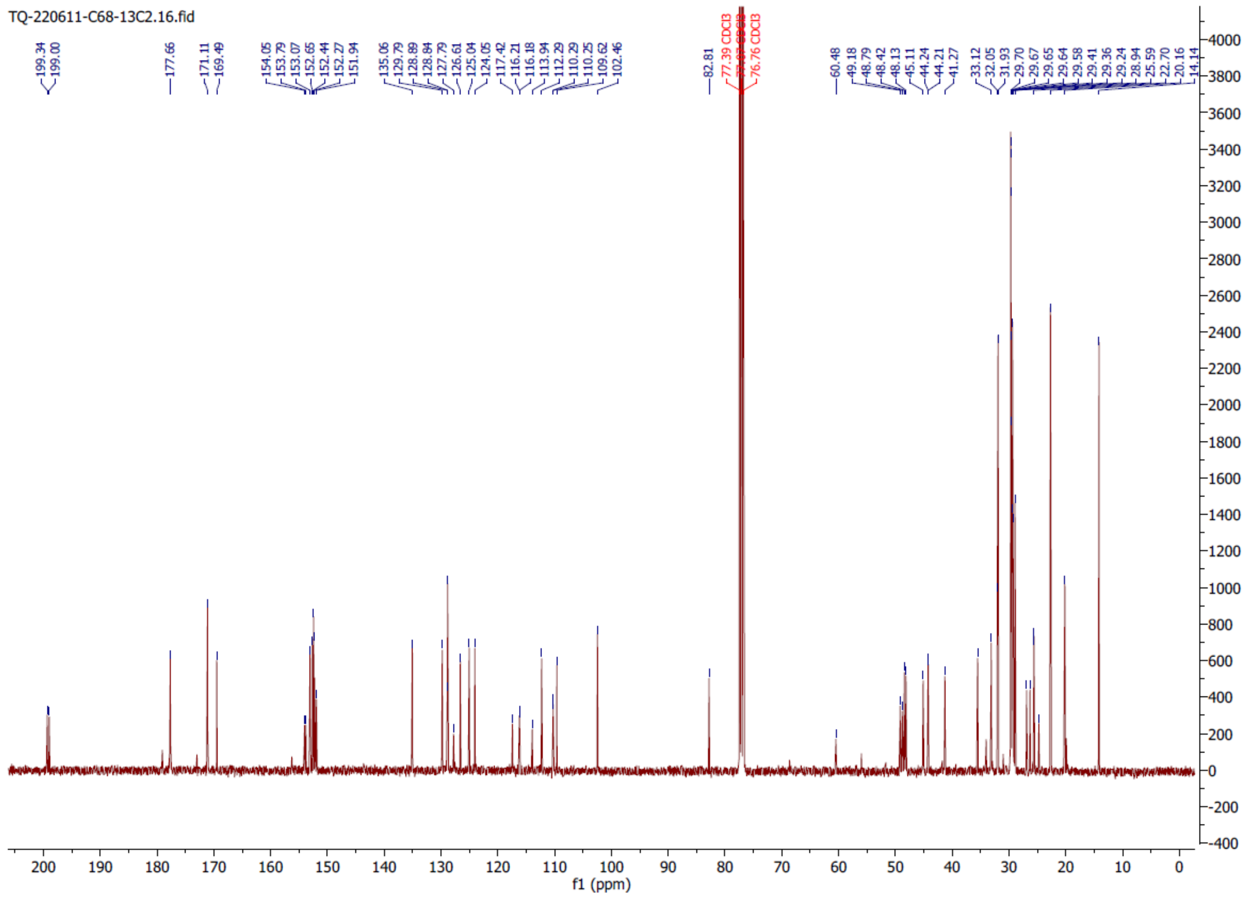

PTP-3

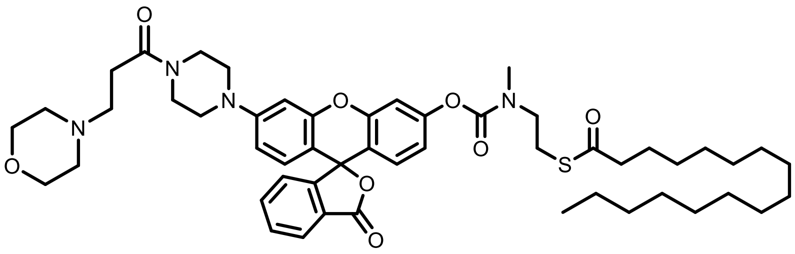

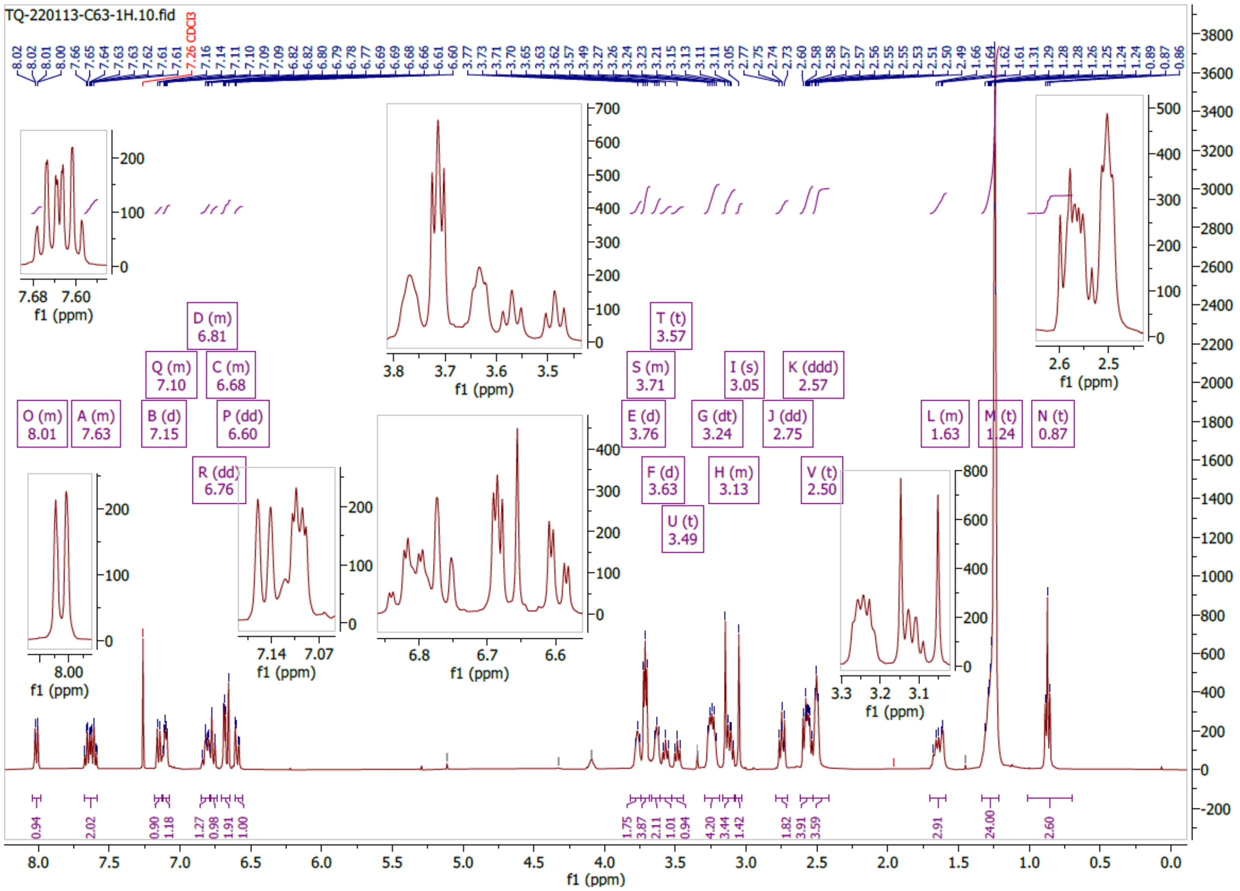

TQ-220113-C63-1H.10.fid
CDCl3
O (m) 8.01
A (m) 7.63
B (d) 7.15
Q (m) 7.10
D (m) 6.81
R (dd) 6.76
C (m) 6.68
P (dd) 6.60
E (d) 3.76
S (m) 3.71
F (d) 3.63
T (t) 3.57
U (t) 3.49
G (dt) 3.24
H (m) 3.13
I (s) 3.05
J (dd) 2.75
K (ddd) 2.57
V (t) 2.50
L (m) 1.63
M (t) 1.24
N (t) 0.87
f1 (ppm)

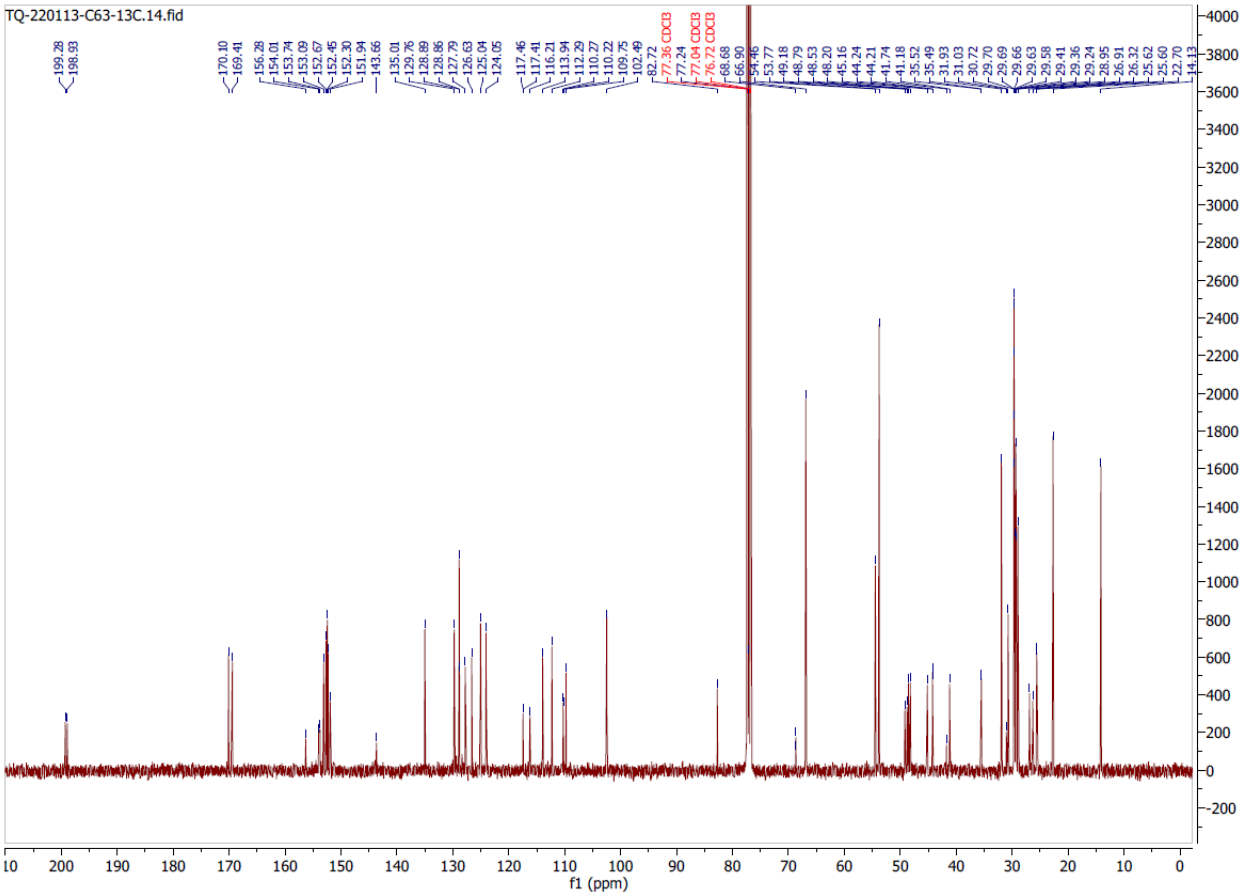
TQ-220113-C63-13C.14.fid
199.28
198.93
170.10
169.41
156.28
154.01
153.74
153.09
152.67
152.45
152.30
151.94
143.66
135.01
129.76
128.89
128.86
127.79
126.63
125.04
124.05
117.46
117.41
116.21
113.94
112.29
110.27
110.22
109.75
102.49
82.72
77.36 CDCl3
77.24
77.04 CDCl3
76.72 CDCl3
68.68
66.90
54.46
53.77
49.18
48.79
48.53
48.20
45.16
44.24
44.21
41.74
41.18
35.52
35.49
31.93
31.03
30.72
29.70
29.69
29.66
29.63
29.58
29.41
29.36
29.24
28.95
26.91
26.32
25.62
25.60
22.70
14.13
f1 (ppm)

Compound 9

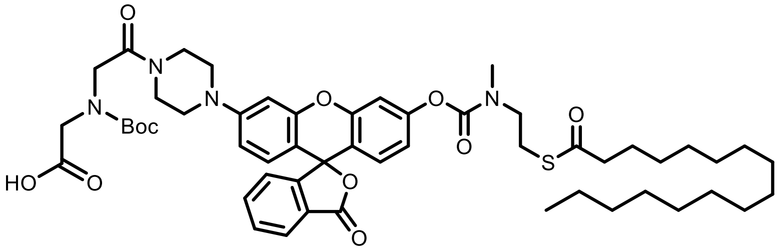

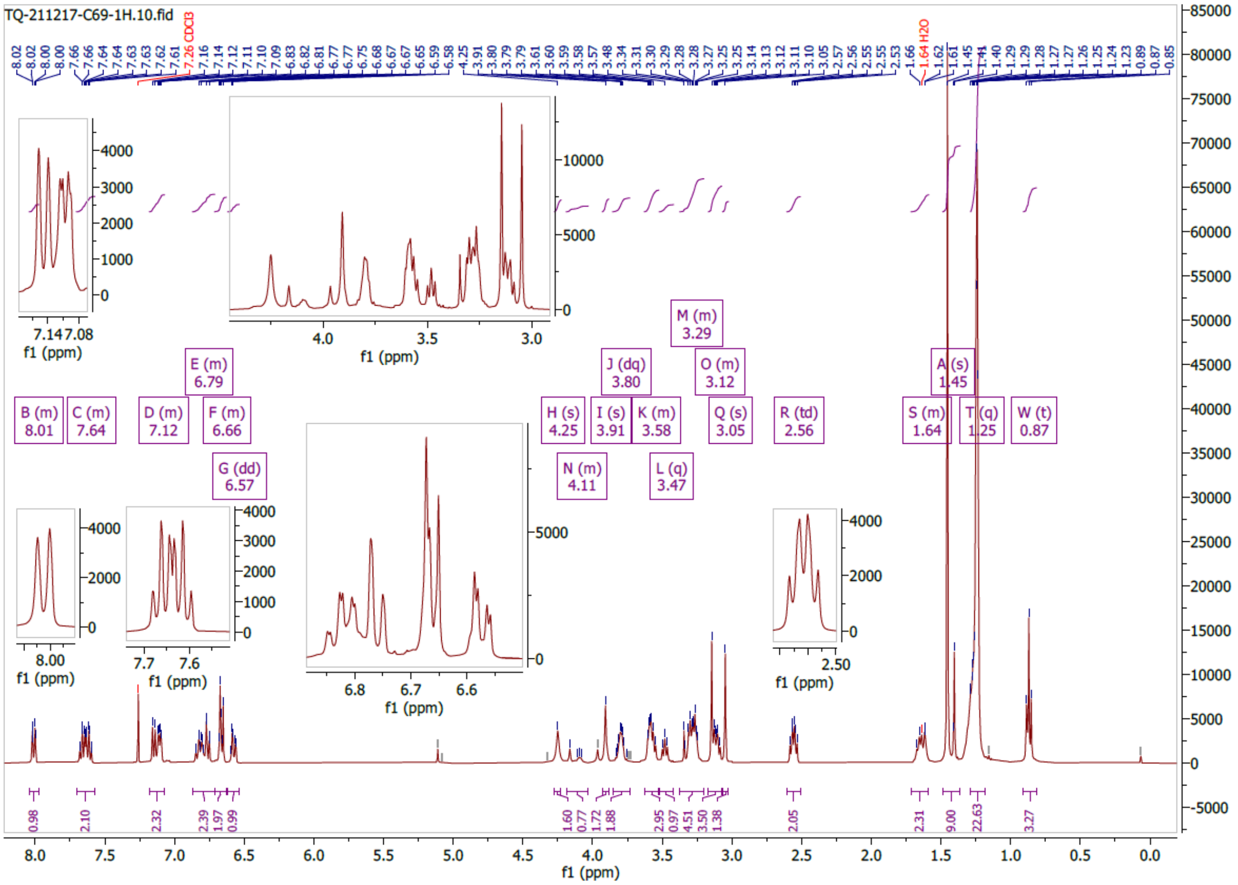
TQ-211217-C69-1H.10.fid
B (m) 8.01
C (m) 7.64
D (m) 7.12
E (m) 6.79
F (m) 6.66
G (dd) 6.57
H (s) 4.25
N (m) 4.11
I (s) 3.91
J (dq) 3.80
K (m) 3.58
L (q) 3.47
M (m) 3.29
O (m) 3.12
Q (s) 3.05
R (td) 2.56
S (m) 1.64
A (s) 1.45
T (q) 1.25
W (t) 0.87
f1 (ppm)

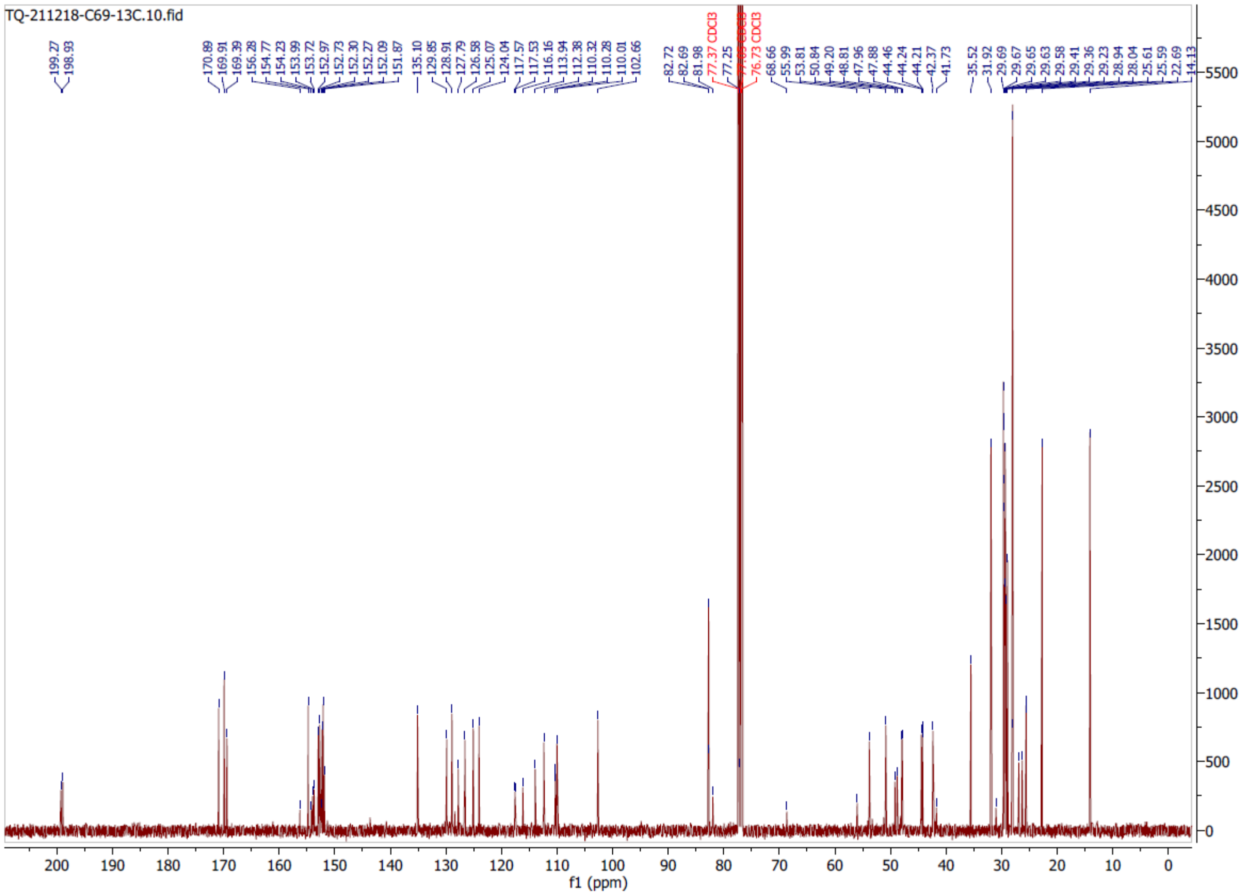
TQ-211218-C69-13C.10.fid
f1 (ppm)

PTP-4

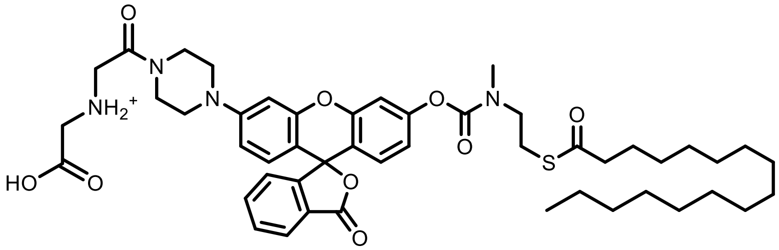

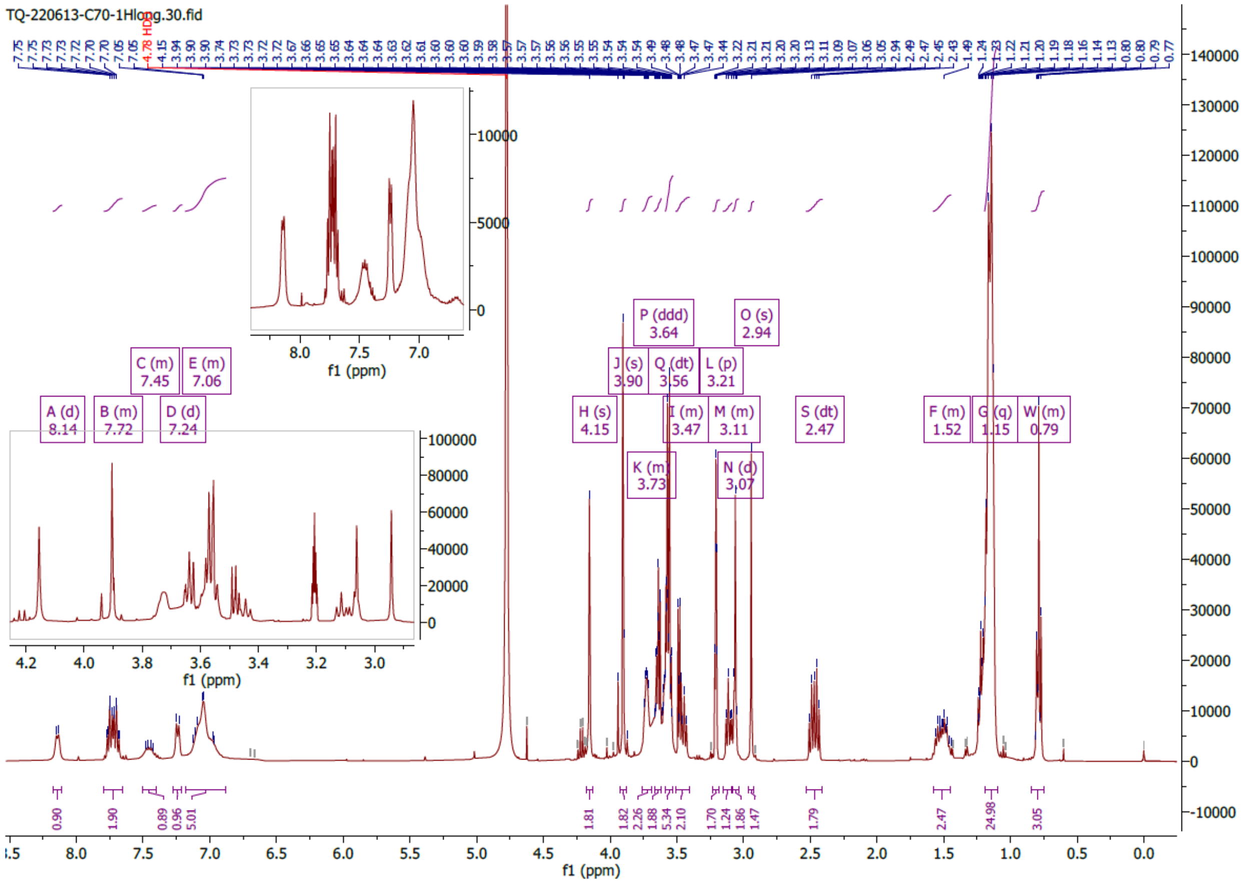
TQ-220613-C70-1Hlong.30.fid
A (d) 8.14
B (m) 7.72
C (m) 7.45
D (d) 7.24
E (m) 7.06
H (s) 4.15
J (s) 3.90
K (m) 3.73
P (ddd) 3.64
Q (dt) 3.56
I (m) 3.47
L (p) 3.21
M (m) 3.11
N (d) 3.07
O (s) 2.94
S (dt) 2.47
F (m) 1.52
G (q) 1.15
W (m) 0.79
f1 (ppm)

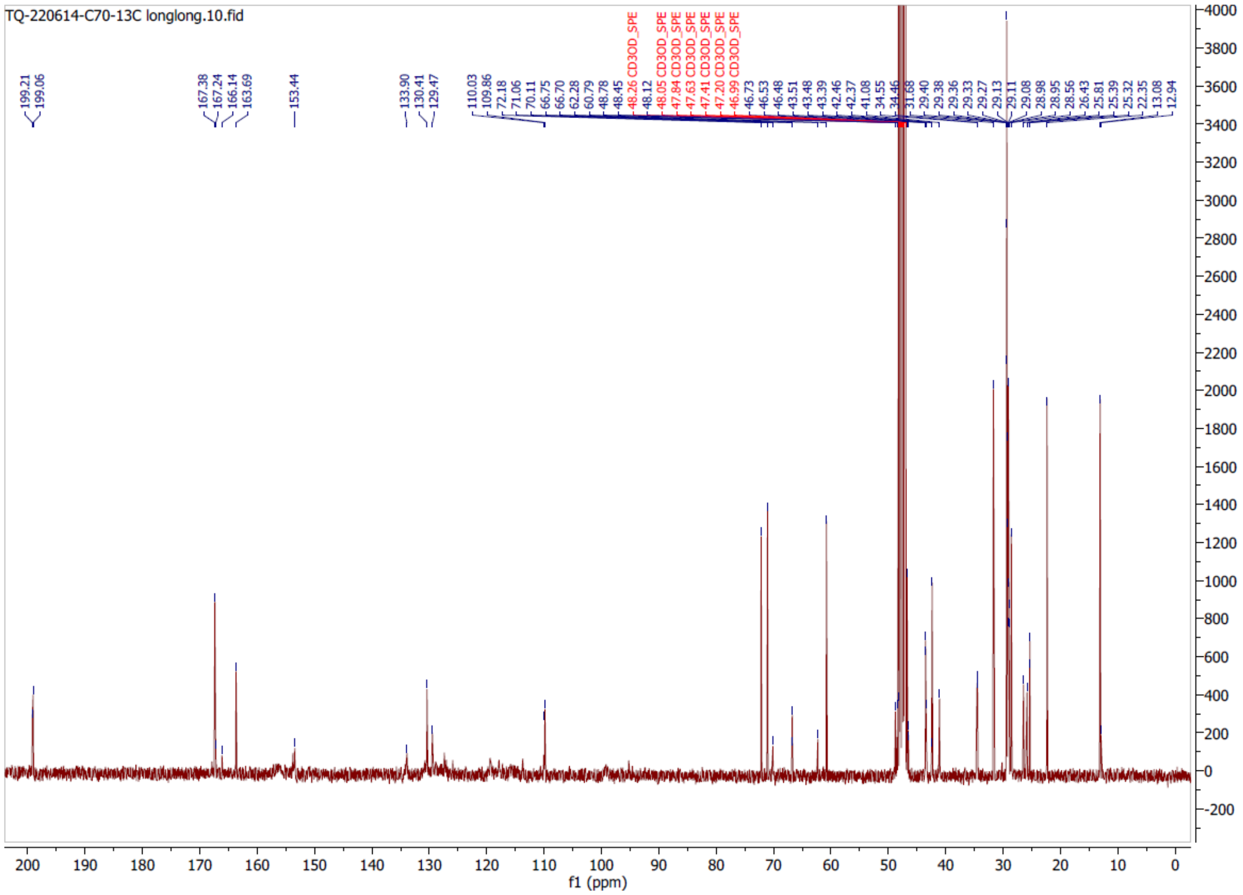
TQ-220614-C70-13C longlong.10.fid
f1 (ppm)

REFERENCES

1. Rana, M. S.; Kumar, P.; Lee, C. J.; Verardi, R.; Rajashankar, K. R.; Banerjee, A., Fatty acyl recognition and transfer by an integral membrane S-acyltransferase. *Science* **2018,** *359* (6372).

2. Azizi, S. A.; Lan, T.; Delalande, C.; Kathayat, R. S.; Banales Mejia, F.; Qin, A.; Brookes, N.; Sandoval, P. J.; Dickinson, B. C., Development of an Acrylamide-Based Inhibitor of Protein S-Acylation. *ACS Chem Biol* **2021,** *16* (8), 1546-1556.

3. Eberhardt, J.; Santos-Martins, D.; Tillack, A. F.; Forli, S., AutoDock Vina 1.2.0: New Docking Methods, Expanded Force Field, and Python Bindings. *J Chem Inf Model* **2021,** *61* (8), 3891-3898.

4. Pettersen, E. F.; Goddard, T. D.; Huang, C. C.; Couch, G. S.; Greenblatt, D. M.; Meng, E. C.; Ferrin, T. E., UCSF Chimera--a visualization system for exploratory research and analysis. *J Comput Chem* **2004,** *25* (13), 1605-12.

5. Malgapo, M. I. P.; Linder, M. E., Purification of Recombinant DHHC Proteins Using an Insect Cell Expression System. *Methods Mol Biol* **2019,** *2009*, 179-189.

6. Kathayat, R. S.; Cao, Y.; Elvira, P. D.; Sandoz, P. A.; Zaballa, M. E.; Springer, M. Z.; Drake, L. E.; Macleod, K. F.; van der Goot, F. G.; Dickinson, B. C., Active and dynamic mitochondrial S-depalmitoylation revealed by targeted fluorescent probes. *Nat Commun* **2018,** *9* (1), 334.
